# Bioinformatics in Sudan: Status and challenges case study: The National University-Sudan

**DOI:** 10.1371/journal.pcbi.1009462

**Published:** 2021-10-21

**Authors:** Sofia B. Mohamed, Sumaya Kambal, Sabah A. E. Ibrahim, Esra Abdalwhab, Abdalla Munir, Arwa Ibrahim, Qurashi Mohamed Ali

**Affiliations:** Bioinformatics and Biostatistics Department, National University Biomedical Research Institute, National University-Sudan, Khartoum, Sudan; McGill University, CANADA

## Abstract

The ever increasing applications of bioinformatics in providing effective interpretation of large and complex biological data require expertise in the use of sophisticated computational tools and advanced statistical tests, skills that are mostly lacking in the Sudanese research community. This can be attributed to paucity in the development and promotion of bioinformatics, lack of senior bioinformaticians, and the general status quo of inadequate research funding in Sudan. In this paper, we describe the challenges that have encountered the development of bioinformatics as a discipline in Sudan. Additionally, we highlight on specific actions that may help develop and promote its education and training. The paper takes the National University Biomedical Research Institute (NUBRI) as an example of an institute that has tackled many of these challenges and strives to drive powerful efforts in the development of bioinformatics in the country.

## Author summary

Bioinformatics is gaining recognition globally and in Sudan as an important subdiscipline of biological sciences, one that enables researchers to efficiently interpret complex biological data. A limited number of Sudanese academic institutions have acknowledged this field despite its increasingly recognized importance. The development of bioinformatics in the country requires interdisciplinary collaborations involving experts in life sciences, research methodology, healthcare, computer, and data sciences. This can be achieved through designing educational programs and workshops alongside proposing and establishing effective collaborative research projects. In this context, we comprehensively discussed the present state of bioinformatics in Sudan, the challenges faced, as well as the efforts exerted by academic institutions including NUBRI, to upgrade infrastructure and establish local and international collaborations.

## Introduction

Advances in bioinformatics continue to enable interdisciplinary research teams to handle, analyze, and interpret large biological datasets. Technological advances in genome sequencing and computational capacities have provided deeper insights into various fields including biomedical sciences, healthcare, and agriculture, among others [1]. The emergence of bioinformatics in Africa was a response to the development of initiatives aimed at establishing strong bioinformatics capacities across the continent. In 1996, Winston Hide recognized the need for bioinformatics development when he founded the South African National Bioinformatics Institute (SANBI, http://www.sanbi.ac.za/) at the University of the Western Cape (UWC) [2]. Seven years later, several initiatives and training nodes were established in South Africa, Kenya, Tunisia, and Sudan, with the aim of providing training programs and workshops for African researchers and postgraduate bioinformatics students. In 2004, the African Society for Bioinformatics and Computational Biology (ASBCB) was established as a dedicated society for bioinformatics development in Africa [3]. Following its first conference in Kenya in 2007, the ASBCB has been running biannual meetings accompanied by training workshops in partnership with the International Society of Computational Biology (ISCB) [4]. These have introduced students and researchers to a wide range of exciting opportunities in the field. The 2 most pivotal events, which occurred in 2010, were the establishment of Human Heredity and Health in Africa initiative (H3Africa, http://www.h3africa.org) and the development of a pan-African bioinformatics network (H3ABioNet, https://www.h3abionet.org/) in response to gaps in human genomic research in African populations and to develop bioinformatics capacity in the continent, respectively [5]. Recently, and despite the challenges to be described later, there has been notable progress in the development of bioinformatics in the African continent. This is manifested in the rising number of initiatives, research funding, degree programs (BSc, MSc, and PhD), internships, and training opportunities. It can be claimed that bioinformatics education in Africa has been revitalized through H3ABioNet initiatives since 2010. As a result, various groups of bioinformaticians and wet lab researchers have become increasingly interested in workshops and academic programs in this field. These efforts, in addition to published studies on the development of bioinformatics in several countries [6,7], have inspired us to investigate the status of bioinformatics in Sudan. We attempted to investigate the current situation of bioinformatics education and research in the country through reviewing the number of Sudanese bioinformatics publications, studying the outcomes of genomics and bioinformatics research projects in national institutions, thereby examining the effects of the shortage of trained bioinformaticians, and exploring logistics and infrastructural limitations. The challenges and proposed solutions have been discussed in the light of our own initiatives at the National University-Sudan (NUSU).

## Bioinformatics in Sudan

### Sudanese bioinformatics institutions

As of 2021, there are 4 Sudanese institutions that offer bioinformatics data analysis and education: (1) the Centre for Bioinformatics and Systems Biology (CBSB), University of Khartoum; (2) the Department of Molecular Biology and Bioinformatics at the College of Veterinary Medicine, University of Bahri, Khartoum; (3) the Department of Applied Bioinformatics and Genomics at the Africa City of Technology; and (4) the Department of Bioinformatics and Biostatistics at the National University Biomedical Research Institute (NUBRI), NUSU.

### Centre for bioinformatics and systems biology

The CBSB is one of the H3ABioNet nodes in Africa [8], established in 2014. It has been involved in many bioinformatics, computational biology, and human genomics research. It has also contributed to training, education, and building of bioinformatics capacities, as well as providing a guide to users in selecting suitable DNA sequence stimulation tools. This is in addition to providing updates on current trends, challenges, and opportunities behind established next-generation sequencing (NGS) simulation tools [9]. It has addressed these challenges by presenting a blended learning bioinformatics course and webinar series in a developing country with limited resources. These series have been developed from experiences gained from hosting H3ABioNet events (courses, workshops, webinars, etc.) [8]. It has also collaborated in the running of many hackathons that aim at sharing new skills and experience among African scientists studying the encounters between the participants as well as the outcomes, including H3ABioNet Cloud Computing and Malaria Hackathons [10]. In addition, the CBSC has investigated the Swift/Tl workflow management system by applying a genomic variant calling workflow to Swift/T Language [11].

### Department of Molecular Biology and Bioinformatics, College of Veterinary Medicine, University of Bahri, Khartoum

This department provides bioinformatics training in addition to PhD and MSc degrees in molecular biology and bioinformatics. It has been primarily involved in vaccine design and human genetics. These included the prediction of multiepitope vaccines for Peste des Pettis Ruminants virus [12] and avian infectious laryngotracheitis virus [13]. Likewise, in-silico and computational examination was used in many studies that evaluate the impacts caused by different SNPs like SNPS in the human *PRAG* gene [14] and novel deleterious non-synonymous SNPs within the *MEFV* Gene [15] and the most deleterious nsSNPs in the human *GGCX* gene [16].

### Department of Applied Bioinformatics and Genomics, Africa City of Technology, Khartoum

Established in 2007, the Department of Applied Bioinformatics and Genomics is one of the departments that make up Africa City of Technology, a research institute located in Khartoum North, Sudan. It provides workshops and training courses in bioinformatics, advanced genomics, and molecular genetics research. One research area investigated by the department was breast cancer pathogenic variants, particularly *BRCA2* gene nonsense mutations in Sudanese women [17]. Bioinformatics tools were also used to study other genetic diseases such as Merosin-deficient congenital muscular dystrophy [18] and nonsyndromic hearing impairment in Syrian patients [19]. These 2 projects were conducted as part of an international collaboration with the Faculty of Biotechnology, University of Malakand, Pakistan and the Department of Neurology and Epileptology, Hertie Institute for Clinical Brain Research, Tuebingen, Germany.

### Department of Bioinformatics and Biostatistics, National University-Sudan

A relatively recent addition to the NUBRI, the Department of Bioinformatics and Biostatistics, has been conducting research, providing workshops, presentations, and training courses since 2017. Applications in genomics, proteomics, structural biology, drug discovery, computational biology, and data science are all explored as part of the department’s work. Pathogen genomics, however, is the main focus of past and ongoing research projects. An example is the whole genome sequencing of gram-negative bacteria and gram-positive bacteria isolated from Sudanese patients including *Escherichia coli* [20], *Pseudomonas aeruginosa* [21], and Methicillin-Resistant *Staphylococcus aureus* (MRSA) [22]. Furthermore, a proteomics and molecular docking study performed on MRSA strains can provide insights into combating β-lactam–resistant bacteria in the future [23].

## Challenges to the growth of bioinformatics in Sudan

Although bioinformatics has become well established in some parts of Africa, it can still be considered to be in its formative stages in Sudan. This is attributed to many challenges including the lack of access to local bioinformatics expertise and training, internet availability, speed, and instability and the lack of laboratory infrastructure. These difficulties have hindered bioinformatics development and education. Additionally, until recently, Sudan has faced strict economic sanctions that have obstructed funding and the possibility of international collaborations with reputable institutions. These restrictions have also included access to a number of advanced massive open online courses (MOOCs) in molecular biology and bioinformatics. The expected relief from sanctions, although widely reported in local and international media, is yet to be fully implemented. The advent of the Coronavirus Disease 2019 (COVID-19) pandemic has further crippled an already struggling economy. Despite this, enthusiasm for bioinformatics among Sudanese researchers has only increased in recent years as a result of ongoing workshops and courses. This, however, has not been corroborated by the establishment of a sufficient number of formal bioinformatics degree programs at both the undergraduate and graduate levels. The analyses of large datasets generated from bioinformatics pipelines requires the establishment of a bioinformatics laboratory. There are difficulties, however, in setting up such facilities, even with the availability of experts with the required skills and experience. Therefore, expanding this computing and IT infrastructure is a priority. It will allow for the wide scale application of bioinformatics in Sudan. Moreover, the availability of technical facilities will put Sudan in line with global institutions in terms of capacities and will lead to more effective collaborations, the enhanced sharing of data, and, eventually, the translation of these research and academic efforts into science and clinical practice.

### Publications in bioinformatics

The bioinformatics community in Sudan has been steadily growing, with researchers from various scientific backgrounds and diverse interests, including pathogen genomics, human genetics, computer science, plant, and animal genetics. The databases PubMed and ScienceDirect [24] were searched from the period between 2003 and 2020 using the following query terms: “next-generation sequencing,” “computational biology,” “bioinformatics,” genomics,” and “in silico” as mentioned by authors [25]. Another search term, “sequencing,” was added to include publications that used first generation sequencing or Sanger sequencing. After discarding duplicate papers, all publications were filtered according to the following inclusion and exclusion criteria.

#### Inclusion criteria

The selection included articles written in English, authored by at least 1 scientist affiliated with a Sudanese institution and incorporating bioinformatics techniques according to the definition by authors [26].

#### Exclusion criteria

Some publications were excluded from the study like those affiliated with South Sudan (an independent country since 2011). Also excluded were publications that were available only as abstracts, where we were not able to confirm whether a bioinformatics tool had been used, and reviews (40 papers), due to the lack of a bioinformatics tool application.

### Publications data analysis

**Fig 1:**
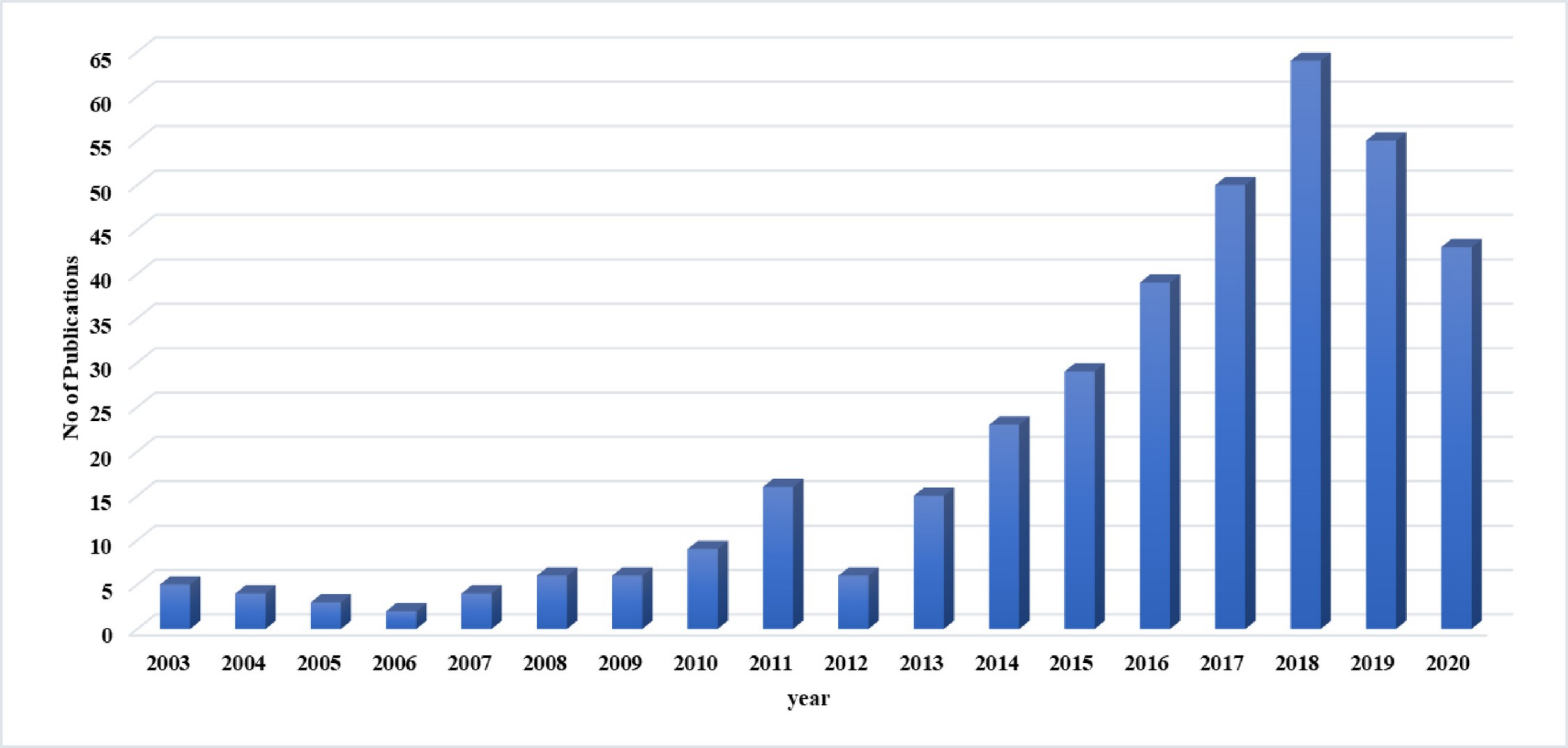
Bioinformatics-related publications in Sudan. The diagram indicates the number of hits obtained in a search of PubMed and ScienceDirect using the terms “next-generation sequencing,” “computational biology,” “bioinformatics,” “genomics,” “in silico,” and “sequencing” filtered by authors affiliated with Sudanese institutions from 2003 to 2020.

**Fig 2:**
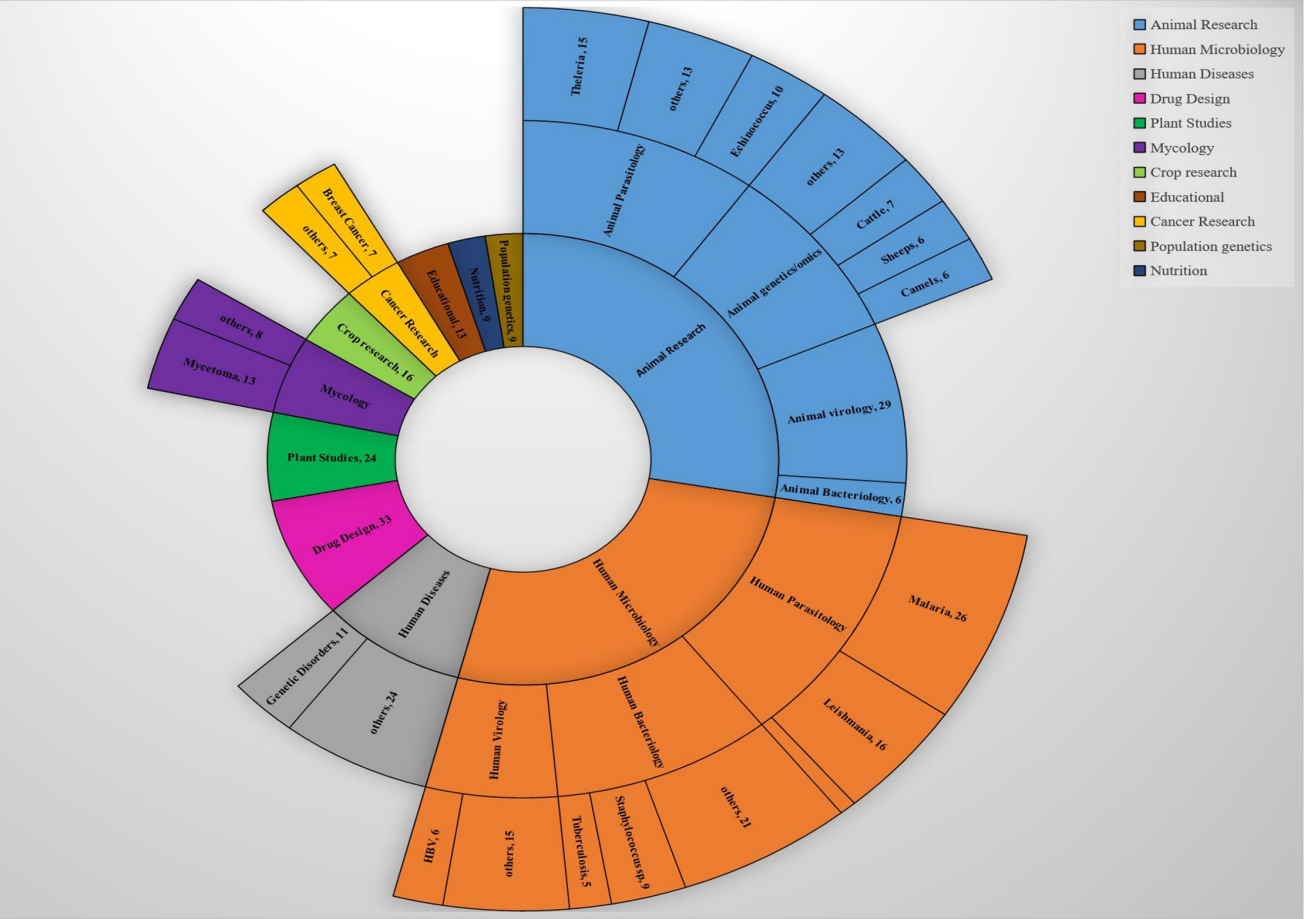
Research areas covered by Sudanese scientists in bioinformatics-related publications. The figure describes all research areas, subresearch areas, and topics retrieved from a search of PubMed and ScienceDirect using the terms “next-generation sequencing,” “computational biology,” “bioinformatics,” “genomics,” “in silico,” and “sequencing” filtered by authors affiliated with Sudanese institutions. All the numerical data used in all figures are included in S1 Data.

The final outcome was 379 articles (Fig 1) (S1 Table) (retrieved on 10-5-2020). The chosen articles included those with methodologies that incorporate bioinformatics techniques, such as genome/exome sequencing, sequence alignment, phylogenetic trees, primer design, single nucleotide polymorphism (SNP) analysis, and more. The present study has indicated that bioinformatics tools and software have been used to investigate a number of research areas in Sudan as illustrated in Fig 2. They have mostly focused on animal research and human microbiology (malaria, leishmania, theileria, mycetoma, echinococcus, and staphylococcus), genetics, and genomics, and, later, human disease, drug design, plant studies, crop research, mycology, cancer research, population genetics, and human nutrition. The articles relating to subjects from Sudan are usually collaborative projects between scientists in Sudanese institutions and those in regional or international research centers. These projects include studies that have analyzed the full genome sequence of Dromedary camels to evaluate the genomic diversity and signatures of positive selection [27], the phylogenetic tree analysis of Streptomyces isolates from soil [28], and anti-hepatitis B virus activity isolated from *Guiera senegalensis* leaves [29]. The studies also included articles that investigated the prediction of the appropriate Middle East Respiratory Syndrome Coronavirus (MERS-CoV) epitope vaccine [30], identification of mutations in the *Theileria annulata* prolyl isomerase I gene (*TaPIN1*) [31], malaria drug resistance molecular markers [32], whole genome sequencing of *Klebsiella pneumoniae* [33] and *Acinetobacter baumannii* [34], and the function of EIL/EIN3 transcription factor genes in cotton fiber development [35].

## Development of Bioinformatics in National University-Sudan

Despite the abovementioned challenges, the NUBRI has been trying since 2017 to overcome all of these obstacles and lead efforts to advance bioinformatics applications and learning in the country. The institute is continuing to offer dependable solutions for research problems through the analysis and interpretation of biological data. The Department of Bioinformatics and Biostatistics at NUBRI is focused on using a myriad of bioinformatics tools in the adequate computational and IT infrastructure, including network and storage systems. The research team is comprised of experienced researchers with the skills to analyze bioinformatics pipelines in addition to wet lab methodologies and techniques. In this section we describe NUBRI’s efforts in the development of bioinformatics.

### The mission and objectives of the bioinformatics and biostatistics department

#### Mission

The mission of this department is to (a) conduct bioinformatics and computational biology research relevant to Sudan; (b) develop human resources in bioinformatics and computational biology by educating and mentoring scientists; and (c) raise awareness and access to bioinformatics and computational biology resources.

#### Objectives

The objectives of the department are to (a) generate and publish high quality biomedical research; (b) train competent researchers; (c) upgrade the academic programs of the NUSU; (d) enhance other research fields through collaborative projects; and (e) establish sources of sustainable funding to realize the missions of NUBRI.

#### Infrastructure

One of the key challenges to the application of bioinformatics in Sudan is the lack of reliable access to a stable internet connection, which is key to accessing web-based bioinformatics tools and even client tools often require access to online datasets. Another more significant obstacle is the instability of the electricity supplied to educational institutions. NUBRI has been able to overcome these challenges as a result of the availability of substantial technical, logistical, and financial resources, which have made its designated objectives attainable. The institute has invested in obtaining the necessary software and hardware for networking, storage, and data analysis to enable efficient processing of sequence data. These facilities are also comprised of a 24-hour power supply, robust internet connections, and a bioinformatics lab supplied with high-performance computers. The university has provided a server with high processing power, memory, and storage (2 × 12 Intel Xeon CPU, 350 GB DDR4 RAM, 10 TB SSD HD) that is suitable for massive data storage and computing resources.

#### Training capacity

Basic bioinformatics training programs are highly necessary to increase bioinformatics capacities. NUBRI has provided those opportunities with an ultimate aim of enhancing the development of bioinformatics in Sudan. It is committed to deliver a number of short training courses, available both on-site and online, throughout the year. This has supported students in their MSc and PhD programs in bioinformatics-related disciplines. Below is a brief description of the activities and programs offered at the institution:

1. **Short-term training courses and workshops:** These are intended to familiarize students and those interested in the field with a wide range of bioinformatics topics including databases, DNA sequence analysis, and genome browsing. Table 1 illustrates workshops and training programs held at NUBRI between 2018 and 2019. They were structured in a way to engage individuals of diverse academic backgrounds. The majority of these workshops were accompanied with hands-on activities to enable participants to handle real data and bioinformatics tools. In order to continuously improve the quality of the workshops offered by the institute, participants were invited to fill in evaluation forms at the end of each workshop. They were also given handouts and suggestions for further reading and practice to ensure that they are able to apply what they have learned.

**Table 1:** Workshops held at the NUBRI.

No.	Workshop	Date	Fund
**1**	Introduction to Bioinformatics	11/9 to 11/12-2019	Pan African Bioinformatics Network H3ABioNet, South Africa/NUSU
**2**	African Genomic Medicine Training for Nurses Initiative (AGMT)	13/3 to 4/7-2019	Pan African Bioinformatics Network H3ABioNet, South Africa/NUSU
**3**	Principal investigator in workshop of 16S rRNA Intermediate Bioinformatics Training (Int_BT)	9/8 to 10/10-2019	Pan African Bioinformatics Network H3ABioNet, South Africa/NUSU
**4**	Introduction to Bioinformatics	11/9 to 27/12-2018	Pan African Bioinformatics Network H3ABioNet, South Africa/NUSU
**5**	Investigation of non-coding yeast mitochondrial genome variation via experimental evolution	20/11/2018	NUSU
**6**	Research Methodology	21-22/10-2018	NUSU
**7**	The 3rd International Symposium and Workshop on Functional Genomics and Structural Biology	23-27/07-2018	NUSU/UPM
**8**	Workshop on Bioinformatics, Drug Discovery and Molecular Simulation Dynamics	24-27/04-2018	NUSU, in collaboration with the BioDiscovery Group, India
**9**	R-Statistics	10-20/9-2017	
**10**	Basic Bioinformatics	22-27/1-2017	NUSU
**11**	R-Statistics	7-21/1-2017	NUSU
**12**	Basic Molecular Biology and Bioinformatics	29/10-29/12-2017	NUSU

NUBRI, National University Biomedical Research Institute; NUSU, National University-Sudan; UPM, Universiti Putra Malaysia.
2. **Research Attachment Program (RAP):** In 2018, RAPs were established to train students in one of the subdisciplines of bioinformatics. During their 2 to 3 months at the Department of Bioinformatics and Biostatistics, students are given the opportunity to work in projects, analyze sequencing data, and participate in writing a scientific publication. This program focuses on training participants to use bioinformatics data packages and web-based interactive tools to analyze real data generated from the department’s research projects. Between 2018 and 2020, the program has recruited 50 researchers from various disciplines including biotechnology, pharmacy, molecular biology, laboratory technology, veterinary medicine, human medicine, and dentistry.
3. **Classrooms for H3ABioNet workshops:** In 2017, the department succeeded in hosting 1 of 20 classrooms of the Pan African Bioinformatics Network for H3Africa (H3ABioNet). The department has so far hosted classrooms for 3 introductory level courses as illustrated in the Table 1. These 3-month courses are offered free of charge without application or participation fees. This has enabled participants from local universities and major research institutions to gain access to African expertise and bioinformatics trainers via video conferencing and discussion forums [36].
4. **Staff training:** Researchers at NUBRI are encouraged to regularly attend training workshops to ensure that they possess the necessary skills and knowledge in order to effectively collaborate with regional and global bioinformatics institutes. This has been accomplished through providing bursaries and travel support. Trained staff, in turn, act as trainers and arrange courses to transfer skills to other university staff. Table 2 summarizes the participation of NUBRI staff at conferences and workshops worldwide.

**Table 2:** International workshops and conferences in which NUBRI staff have participated.

No.	Conferences and workshops	Date	Institution	Fund
**1**	Resources and Tools for Computer-Aided Drug Discovery	15/11/2019	ISCB Africa ASCB conference on Bioinformatics, Kwame Nkrumah University of Science and Technology (KNUST) Ghana	NUSU
**2**	H3ABioNet-Data Management Plans for Grant Proposals and Research	15/11/2019		
**3**	Galaxy Africa	13-14/11-2019		
**4**	New Features of the UCSC Genome Browser	13-14/11-2019		
**5**	Train the Trainer: Capacity Building for Genomic Surveillance of AMR in low- and middle-income countries	6-13/10-2019	Wellcome Genome Campus, United Kingdom	Wellcome Genome Campus/NUSU
**6**	Next Generation Sequencing and Bioinformatics Training Workshop	26/1-2/2-2019	Wellcome Connecting Science (University of Witwatersrand, Johannesburg, South Africa)	Wellcome Connecting Science/NUSU
**7**	The 3rd International Symposium and Workshop on Functional Genomics and Structural Biology	23-27/7-2018	UPM	NUSU
**8**	Working with Pathogen Genomes	17-22/6-2018	Cape Town University—South Africa	Wellcome Connecting Science
**9**	Bioinformatics, Drug Design and Molecular Simulation Dynamics/ A Molecular Modelling Study	24-28/4-2018	The Second Scientific Conference of the NUSU	NUSU
**10**	Applications of Next-Generation Sequencing in Clinical Microbiology	10-14/3-2018	Sudan University of Science and Technology	NUSU
**11**	Next Generation Sequencing (NGS)—Bacteria Whole Genome Sequencing	8-17/4 to 5/5 (2017)	Centre for Chemical Biology, Universiti Sains Malaysia	NUSU
**12**	Advanced Course on Bacteria Whole Genome Sequencing Analysis.	9-8/5 to 23/6 (2017)	Faculty of Biotechnology and Bimolecular Science, UPM	NUSU

NUBRI, National University Biomedical Research Institute; NUSU, National University-Sudan; UPM, Universiti Putra Malaysia.
5. **E-learning:** NUBRI has created an easily accessible electronic learning platform for diploma and master programs to conduct teaching sessions and assessments. NUBRI is also planning to establish an updateable education platform that facilitates knowledge transfer though lecture videos and hands-on practical exercises. Due to the computer-intensive nature of bioinformatics, using e-learning technologies will benefit both instructor and learners by allowing for the easy preparation of teaching materials and efficient evaluation of students’ progress. As for the learners, it offers them the freedom to learn at their own pace and interact easily with other learners and instructors. Along this line, the department of Bioinformatics and Biostatistics is establishing a server platform for data and resources storage and for computational analyses. The server platform is a key achievement that will enhance international collaborations and provide online, country-, and region-wide training and services.

#### Degree programs

The NUSU is currently the only university in the country that offers a degree program in bioinformatics. There were many attempts by Sudanese universities to introduce bioinformatics into current curricula, which have normally focused on integrating aspects of the discipline into existing life science degree programs. At NUBRI, we have developed courses in a way that conforms to the interests of scientists from both biology and computer science backgrounds. This will certainly help in developing multidisciplinary teams with the ability to utilize their respective basic science knowledge and newly acquired bioinformatics skills to handle complex datasets.

The bioinformatics master’s degree program was established in 2019 and is offered over 12 months. It incorporates 13 coursework modules and a research project running concurrently throughout the program. This master’s degree program equips the students with the following:

1. a basic background in modern biology, biochemistry, cell biology, genetics, and molecular biology;
2. familiarity with computational methods to address problems of digital technology in molecular biology;
3. knowledge on storage, retrieval, sharing, and use of biological information in core areas of bioinformatics: multiple sequence alignment, phylogenetic trees, genomics, and proteomics; and
4. skills in applied bioinformatics: immunoinformatics, drug designing, and discovery.

The program’s courses cover introductory programming and molecular biology before moving on to more advanced topics. The program is evenly divided into coursework and research. The coursework includes diverse modules covering mathematics, statistics, computer science, and biology, with an emphasis on applications to bioinformatics research. While establishing and running the program, a number of challenges arose, such as the required infrastructure and administrative support, range of topics, diversity of students, and finding examiners and program evaluators. The institute actively recruits students from different backgrounds with the belief that diversity positively impacts student interactions and overall academic experience. For purposes of knowledge transfer and promoting collaborations, international experts are invited to provide workshops at the institute in order to keep students updated with all the most recent bioinformatics tools and software used in big data analysis.

Upon successful completion of the master’s program, students should be able to (a) outline strategies and apply appropriate tools in bioinformatics; (b) identify potential bioinformatics applications drawn from ongoing research; (c) interpret data related to sequences of nucleotides and amino acids, protein domains, and protein structures; (d) develop and implement tools that enable efficient access and management of data; (e) acquire essential programming skills; and (f) demonstrate an understanding of the algorithms used in bioinformatics.

In addition to the master’s degree program, diploma and PhD programs are already being developed at NUBRI, as part of its vision to expand and promote the discipline in Sudan.

#### Collaborations

Building bridges among African scientists and non-African scientists is one of the approaches that can help to improve Africa’s participation in bioinformatics and facilitate the development of scientific capacity and productivity in this discipline [16]. At NUBRI, researchers are always encouraged to collaborate and engage with bioinformatics researchers within the country and from various academic institutions around the world such as Universiti Putra Malaysia (UPM), the ISCB, the African Society of Bioinformatics and Computational Biology (ASBCB), the SANBI, and the National Institute for Communicable Diseases (NICD). The institute has collaborative projects with the NICD focused on the field of pathogen genomics. A number of NICD staff were invited to perform workshops and train NUBRI staff at their laboratories in South Africa. In another collaboration with South Africa, one of our staff members joined SANBI as an intern and received bioinformatics training and consequently initiated joint research projects. Partnership agreements with SANBI and other African institutes are currently underway in order to support bioinformatics training in Sudan, through sponsoring conferences, seminars, and internship programs. In 2018, a researcher from NUBRI was trained on NGS analysis at UPM and was successful in promoting collaborations, which is evident by the institute’s contribution in organizing the 3rd International Symposium and Workshop on Functional Genomics and Structural Biology (FGSB 2018) in Malaysia. These networks have contributed to the rapid development of bioinformatics research at the institute as they have provided training opportunities as well as the sharing bioinformatics resources.

#### Projects

As a bioinformatics research group at the NUBRI, pathogen genomics is the core theme of ongoing projects, as shown in Table 3. NGS technology (whole genome sequencing) is being performed to study clinically relevant pathogenic microorganisms. The ultimate aim of these projects is to reduce the health burden of infectious diseases and help in their control. Microbiology screening tests, nucleic acid extraction, and amplification using PCR and reverse transcription PCR (RT-PCR) are carried out in the well-established laboratories of NUSU and NUBRI. Bioinformatics analysis associated with research projects is run by the bioinformatics department at NUBRI using the available server to handle and store large volumes of biological data. In addition, a project aiming at developing a friendly Linux OS for bioinformatics analyses is being developed as illustrated in Table 3.

**Table 3:** Major projects conducted at NUBRI (all are funded by the NUSU).

No.	Project	Period
**1**	Whole Genome Sequencing of Methicillin Resistance *Staphylococcus aureus* Isolated from Khartoum	2017–2018
**2**	Whole Genome Hepatitis Delta Virus (HDV) Among Hepatocellular Carcinoma and Liver Cirrhosis Patients in Khartoum State- Sudan	2020–2022
**3**	Whole-Genome Sequencing of Gram-Negative Bacterial Isolated from Sudan	2019–2020
**4**	Development of Linux System for Bioinformatics Analysis	2019–2020
**5**	Epidemiological Genetic Mapping of Methicillin-Resistant *Staphylococcus aureus* in Sudan’s Hospitals Using Next-Generation Sequencing	2019–2022
**6**	Whole-Genome Sequencing for Detecting Antimicrobial Resistance in Gram-Negative Bacterial Isolated from Sudan	2019–2023
**8**	Molecular Epidemiology of Coronavirus Infection in Sudan	2020–2021
**9**	Whole Genome Sequencing of SARS-CoV-2 Isolated from Alraqi Hospital, NUSU	2021–2022

NUBRI, National University Biomedical Research Institute; NUSU, National University-Sudan.

#### Future strategies

**Fig 3:**
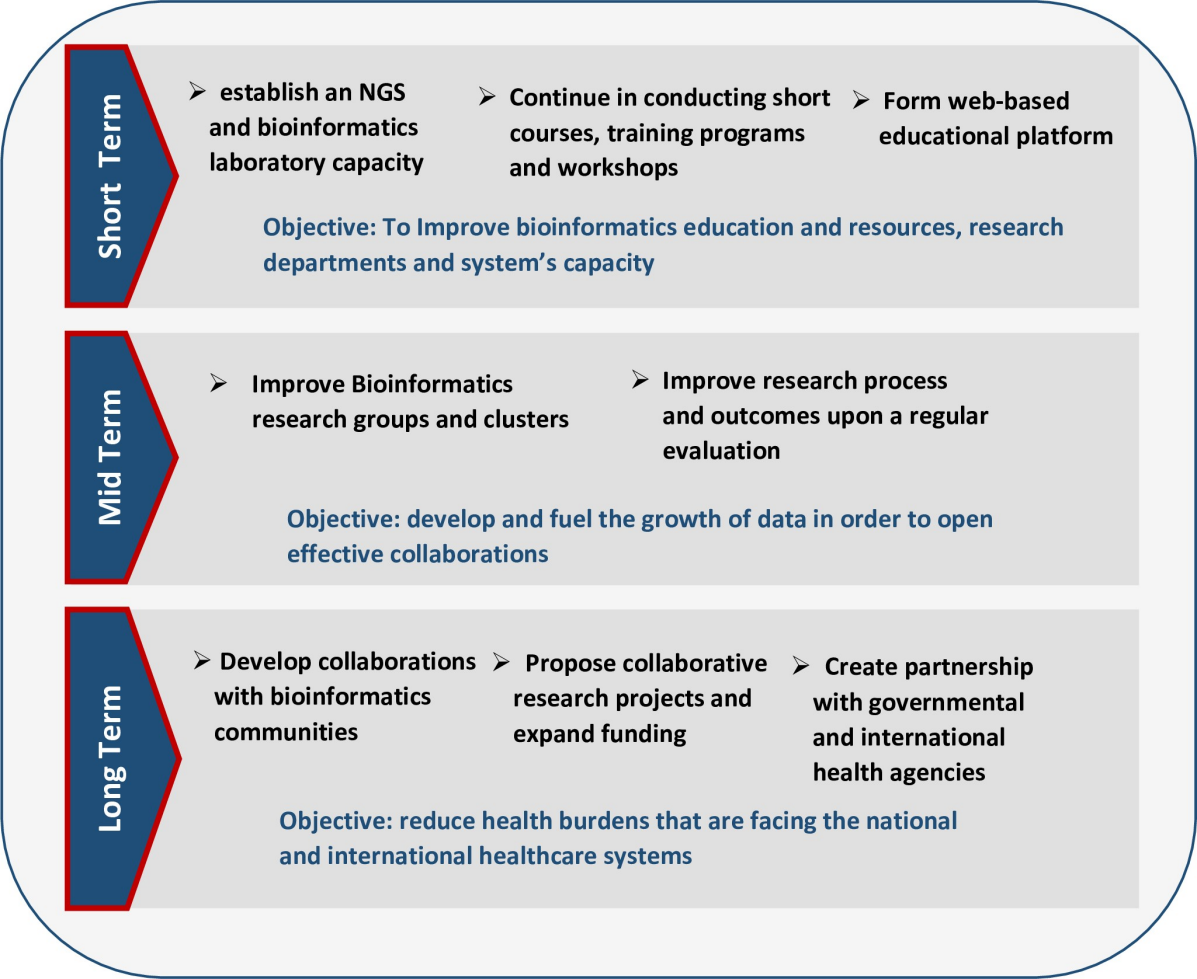
Proposed strategies for promoting bioinformatics in NUBRI. NGS, next generation sequencing; NUBRI, National University Biomedical Research Institute.

The NUSU aims to accelerate the implementation of bioinformatics in the country at the micro level and also the continent at the macro level. This requires a clear road map for dedicated research and support as it has been demonstrated by the South African experience [2]. Fig 3 illustrates our short-, medium-, and long-term strategic plans that focus on providing enhanced educational programs, workshops, resources, scientific research projects, and collaborations. This plan was drafted after carefully following the progress of bioinformatics not only at NUBRI but also at other institutes in Sudan. Regular evaluation and feedback are important aspects of the institute’s plan in order to assess the timeline of achieving goals and provide the resources needed to meet objectives.

#### Short term (1 to 2 years)

At NUBRI, bioinformatics resources and opportunities are always highlighted in discussions and at meetings with researchers, faculty members, policymakers, and even industry leaders. Interactions with researchers at national conferences and symposia have reaffirmed that bioinformatics research development must be a priority in Sudan due to its crucial role and impact on other life sciences. One short-term project currently being established at NUBRI is an NGS laboratory. This is expected to fuel the growth of data and enable the institute to expand research beyond pathogen genomics. A website and web-based educational portal is also in progress. This will provide researchers and students with access to training materials and bioinformatics protocols. Additionally, seminars and conferences are being organized to promote networking not only with educational institutions but also with governmental and industrial entities.

#### Medium term (2 to 4 years)

With the establishment of the bioinformatics and NGS laboratory, NUBRI will be at the forefront of bioinformatics development in the country through the ability to independently sequence project samples and share results nationally and with international research groups. The goal is to collaborate on 3 projects per year by the end of 2024. NUBRI will also focus on securing funds from government-sponsored grants and international funding agencies, taking into account the successful South African experience, which has enabled it to lead bioinformatics research in Africa. The institute will continue in hosting bioinformatics activities and working closely with H3ABioNet, the SANBI, and the NICD. These collaborations will address Africa’s increasing demand for bioinformatics training and research. Implementation of these medium-term strategic goals will also increase Sudan’s contribution to bioinformatics publications in Africa.

#### Long term (4 to 10 years)

In the long term, we plan to conform to nationwide goals set forth by the Sudanese Ministry of Health and WHO, which aim to combat the growing threat of antimicrobial resistance by implementing the National Action Plan (NAP). This action plan was initiated to alleviate the health burden associated with both communicable and noncommunicable diseases in Sudan and Africa and to which bioinformatics is necessary in understanding pathogen genomes and identifying drug targets. At the same time, PhD and postdoctoral training programs are to be established, given that such opportunities will contribute to tackling local problems efficiently and improving the publication records in the country consequently.

## Conclusions

The field of bioinformatics is rapidly evolving and is increasingly being recognized as important to the work of scientists in Africa. In Sudan, interest in life science research and healthcare has created demand for bioinformatics training due to the need to analyze and interpret complex biological data. The field has largely been overlooked previously because of numerous challenges, namely funding and limited local training opportunities. The NUSU and the NUBRI have been leading efforts toward a capacity building plan, detailed in short-, medium-, and long-term goals to support bioinformatics education, training, and research. The institute has succeeded in establishing collaborations, hosting experts in the field, providing training, and has even established a bioinformatics degree program. It is hoped that all these efforts will build even stronger regional and international partnerships and establish Sudan as a key bioinformatics hub in Africa.

## Supporting information

S1 Data Excel spreadsheet containing, in separate sheets, the underlying numerical data for Figs 1 and 2. (XLSX)

S1 Table Bioinformatics-related publications in Sudan from 2003 to 2020. The table indicates the outcome of all the retrieved bioinformatics-related publications authored by Sudanese researchers from 2003 to 2020. The DOI of the comprehensive protocol used in this publication is dx.doi.org/10.17504/protocols.io.bf4njqve.
(PDF)
