# Supplementary material for: Bioinformatics in Sudan: Status and challenges case study: The National University-Sudan
Source: PLoS Comput Biol. 2021 Oct 21;17(10):e1009462. doi: 10.1371/journal.pcbi.1009462 (PMC8530284; doi:10.1371/journal.pcbi.1009462)
Supplement: S1 Table — The table indicates the outcome of all the retrieved bioinformatics-related publications authored by Sudanese researchers from 2003 to 2020. The DOI of the comprehensive protocol used in this publication is dx.doi.org/10.17504/protocols.io.bf4njqve. (PDF) [file pcbi.1009462.s002.pdf]

S1 Table. Bioinformatics-related journal articles published by Sudanese scientists from 2003 – 2020) [a, b]

Year	Title	Journal	Author	Paper link (DOI or PMID)
**2003**	A multiplex PCR for simultaneous detection and differentiation of North American serotypes of bluetongue and epizootic hemorrhagic disease viruses	Comp Immunol Microbiol Infect Dis	I. E. Aradaib, W. L. Smith, B. I. Osburn and J. S. Cullor	10.1016/s0147-9571(02)00035-8
**2003**	Genetic susceptibility to visceral leishmaniasis in The Sudan: linkage and association with IL4 and IFNGR1	Genes Immun	H. S. Mohamed, M. E. Ibrahim, E. N. Miller, C. S. Peacock, E. A. Khalil, H. J. Cordell, J. M. Howson, A. M. El Hassan, R. E. Bereir and J. M. Blackwell	10.1038/sj.gene.6363977
**2003**	Local differentiation in Plasmodium falciparum drug resistance genes in Sudan	Parasitology	A. A. Abdel-Muhsin, M. J. Mackinnon, P. Awadalla, E. Ali, S. Suleiman, S. Ahmed, D. Walliker and H. A. Babiker	10.1017/s0031182003003020
**2003**	Madurella mycetomatis strains from mycetoma lesions in Sudanese patients are clonal	J Clin Microbiol	A. Ahmed, W. van de Sande, H. Verbrugh, A. Fahal and A. van Belkum	10.1128/jcm.41.10.4537-4541.2003
**2003**	Neisseria meningitidis serogroup W-135 isolated from healthy carriers and patients in Sudan after the Hajj in 2000	Scand J Infect Dis	M. Issa, P. Mölling, M. Unemo, A. Bäckman, M. Mosaad, N. Sulaiman and P. Olcén	PMID: 12839149
**2004**	Evolution of drug-resistance genes in Plasmodium falciparum in an area of seasonal malaria transmission in Eastern Sudan	J Infect Dis	A. M. Abdel-Muhsin, M. J. Mackinnon, E. Ali, K. A. Nassir el, S. Suleiman, S. Ahmed, D. Walliker and H. A. Babiker	10.1086/382509
**2004**	Low frequency of deafness-associated GJB2 variants in Kenya and Sudan and novel GJB2 variants	Hum Mutat	N. M. Gasmelseed, M. Schmidt, M. M. Magzoub, M. Macharia, O. M. Elmustafa, B. Ototo, E. Winkler, G. Ruge, R. D. Horstmann and C. G. Meyer	10.1002/humu.9216
**2004**	Molecular epidemiology of canid rabies in Sudan: evidence for a common origin of rabies with Ethiopia	Virus Research	N. Johnson, L. M. McElhinney, Y. H. Ali, I. K. Saeed and A. R. Fooks	https://doi.org/10.1016/j.virusres.2004.04.006
**2004**	SLC11A1 (formerly NRAMP1) and susceptibility to visceral leishmaniasis in The Sudan	Eur J Hum Genet	H. S. Mohamed, M. E. Ibrahim, E. N. Miller, J. K. White, H. J. Cordell, J. M. Howson, C. S. Peacock, E. A. Khalil, A. M. El Hassan and J. M. Blackwell	10.1038/sj.ejhg.5201089
**2005**	International spread of major clones of methicillin resistant staphylococcus aureus: nosocomial endemicity of multi locus sequence type 239 in Saudi Arabia and Romania	Infection, Genetics and Evolution	M. Cîrlan, M. Saad, G. Coman, N. E. Bilal, A. M. Elbashier, D. Kreft, S. Snijders, W. van Leeuwen and A. van Belkum	https://doi.org/10.1016/j.meegid.2004.09.005
**2005**	Serogrouping of United States and some African serotypes of bluetongue virus using RT-PCR	Veterinary Microbiology	I. E. Aradaib, M. E. H. Mohamed, T. M. Abdalla, J. Sarr, M. A. Abdalla, M. A. M. Yousof, Y. A. Hassan and A. R. E. Karrar	https://doi.org/10.1016/j.vetmic.2005.09.014
**2005**	Sudanese mucosal leishmaniasis: isolation of a parasite within the Leishmania donovani complex that differs genotypically from L. donovani causing classical visceral leishmaniasis	Infect Genet Evol	M. Mahdi, E. M. Elamin, S. E. Melville, A. M. Musa, J. M. Blackwell, M. M. Mukhtar, A. M. Elhassan and M. E. Ibrahim	10.1016/j.meegid.2004.05.008

**2006**	A del(X)(p11) carrying SRY sequences in an infant with ambiguous genitalia	BMC Pediatr	M. Ellaithi, D. Gisselsson, T. Nilsson, S. Abd El-Fatah, T. Ali, A. Elagib, M. E. Ibrahim and I. Fadl-Elmula	10.1186/1471-2431-6-11
**2006**	Purification of macroschizonts of a Sudanese isolate of Theileria lestoquardi (T. lestoquardi [Atbara])	Ann N Y Acad Sci	M. A. Bakheit, E. Endl, J. S. Ahmed and U. Seitzer	10.1196/annals.1373.064
**2007**	Analysis on the Origin and Phylogenetic Status of Tong Sheep Using 12 Blood Protein and Nonprotein Markers	Journal of Genetics and Genomics	W. Sun, H. Chang, Z. Yang, R. Geng, K. Tsunoda, Z. Ren, H. Chen and M. H. Hussein	https://doi.org/10.1016/S1673-8527(07)60125-8
**2007**	Comparative assays of the rpoB gene for identification of Mycobacterium tuberculosis isolated from patients in Sudan	Int J Tuberc Lung Dis	A. M. El-Eragi, M. E. Hamid, N. S. Saeed, A. H. Ahmed, K. Lee, Y. H. Kook and M. M. Mukhtar	PMID: 17519100
**2007**	Epidemiological studies on tick-borne diseases of cattle in Central Equatoria State, Southern Sudan	Parasitol Res	D. A. Salih, A. M. El Hussein, U. Seitzer and J. S. Ahmed	10.1007/s00436-007-0583-y
**2007**	IFNG and IFNGR1 gene polymorphisms and susceptibility to post-kala-azar dermal leishmaniasis in Sudan	Genes Immun	M. A. Salih, M. E. Ibrahim, J. M. Blackwell, E. N. Miller, E. A. Khalil, A. M. ElHassan, A. M. Musa and H. S. Mohamed	10.1038/sj.gene.6364353
**2008**	A traditional Sudanese fermented camel's milk product, Gariss, as a habitat of Streptococcus infantarius subsp. infantarius	Int J Food Microbiol	W. Abdelgadir, D. S. Nielsen, S. Hamad and M. Jakobsen	10.1016/j.ijfoodmicro.2008.07.008
**2008**	Comparison of the molecular structure of the TaSP gene of Theileria annulata from Sudanese isolates	Ann N Y Acad Sci	A. M. Ali, D. Salih, M. Bakheit, A. R. El Hussein, S. M. Hassan, M. M. Mukhtar, J. S. Ahmed and U. Seitzer	10.1196/annals.1428.025
**2008**	Evolutionary conservation of RNA editing in the genus Leishmania	Infect Genet Evol	M. E. Ibrahim, M. A. Mahdi, R. E. Bereir, R. S. Giha and C. Wasunna	10.1016/j.meegid.2007.12.009
**2008**	Existence of splicing variants in homologues of Theileria lestoquardi clone-5 gene's transcripts in Theileria annulata and Theileria parva	Ann N Y Acad Sci	M. A. Bakheit, J. S. Ahmed and U. Seitzer	10.1196/annals.1428.023
**2008**	HER-2/neu Ile655Val polymorphism and the risk of breast cancer	Ann N Y Acad Sci	A. Siddig, A. O. Mohamed, H. Kamal, S. Awad, A. H. Hassan, E. Zilahi, M. Al-Haj, R. Bernsen and A. Adem	10.1196/annals.1414.014
**2008**	Identification of Leishmania donovani as a cause of cutaneous leishmaniasis in Sudan	Transactions of the Royal Society of Tropical Medicine and Hygiene	E. M. Elamin, I. Guizani, S. Guerbouj, M. Gramiccia, A. M. El Hassan, T. Di Muccio, M. A. Taha and M. M. Mukhtar	https://doi.org/10.1016/j.trstmh.2007.10.005
**2009**	Capripoxvirus G-protein-coupled chemokine receptor: a host-range gene suitable for virus animal origin discrimination	J Gen Virol	C. Le Goff, C. E. Lamien, E. Fakhfakh, A. Chadeyras, E. Aba-Adulugba, G. Libeau, E. Tuppurainen, D. B. Wallace, T. Adam, R. Silber, V. Gulyaz, H. Madani, P. Caufour, S. Hammami, A. Diallo and E. Albina	10.1099/vir.0.010686-0
**2009**	Distinct haplotypes of dhfr and dhps among Plasmodium falciparum isolates in an area of high level of sulfadoxine–pyrimethamine (SP) resistance in eastern Sudan	Infection, Genetics and Evolution	S. Al-Saai, A. Kheir, A.-M. A. Abdel-Muhsin, A. Al-Ghazali, D. Nwakanma, G. Swedberg and H. A. Babiker	https://doi.org/10.1016/j.meegid.2009.04.010

**2009**	Dubiumin, a chymotrypsin-like serine protease from the seeds of Solanum dubium Fresen	Phytochemistry	I. A. Mohamed Ahmed, I. Morishima, E. E. Babiker and N. Mori	https://doi.org/10.1016/j.phytochem.2009.01.016
**2009**	PCR detection of African horse sickness virus serogroup based on genome segment three sequence analysis	J Virol Methods	I. E. Aradaib	10.1016/j.jviromet.2009.02.012
**2009**	Phenotypic and genotypic characterization of Staphylococcus aureus isolated from raw camel milk samples	Res Vet Sci	E. S. Shuiep, T. Kanbar, N. Eissa, J. Alber, C. Lämmler, M. Zschöck, I. E. El Zubeir and R. Weiss	10.1016/j.rvsc.2008.07.011
**2009**	Phylogenetic analysis of rabies viruses from Sudan provides evidence of a viral clade with a unique molecular signature	Virus Research	D. A. Marston, L. M. McElhinney, Y. H. Ali, K. S. Intisar, S. M. Ho, C. Freuling, T. Müller and A. R. Fooks	https://doi.org/10.1016/j.virusres.2009.07.010
**2010**	A molecular survey of cystic echinococcosis in Sudan	Veterinary Parasitology	R. A. Omer, A. Dinkel, T. Romig, U. Mackenstedt, A. A. Elnahas, I. E. Aradaib, M. E. Ahmed, K. H. Elmalik and A. Adam	https://doi.org/10.1016/j.vetpar.2010.01.004
**2010**	Diversity of lactic acid bacteria from Hussuwa, a traditional African fermented sorghum food	Food Microbiology	N. M. K. Yousif, M. Huch, T. Schuster, G.-S. Cho, H. A. Dirar, W. H. Holzapfel and C. M. A. P. Franz	https://doi.org/10.1016/j.fm.2010.03.012
**2010**	Effect of khat chewing on periodontal pathogens in subgingival biofilm from chronic periodontitis patients	Journal of Ethnopharmacology	N. N. Al-Hebshi, A. K. Al-Sharabi, H. M. Shuga-Aldin, M. Al-Haroni and I. Ghandour	https://doi.org/10.1016/j.jep.2010.08.051
**2010**	Interleukin 10 gene polymorphisms and development of post kala-azar dermal leishmaniasis in a selected sudanese population	Public Health Genomics	S. Farouk, M. A. Salih, A. M. Musa, J. M. Blackwell, E. N. Miller, E. A. Khalil, A. M. Elhassan, M. E. Ibrahim and H. S. Mohamed	10.1159/000272457
**2010**	Molecular characterization of foot-and-mouth disease viruses collected from Sudan	Transbound Emerg Dis	M. Habiela, N. P. Ferris, G. H. Hutchings, J. Wadsworth, S. M. Reid, M. Madi, K. Ebert, K. J. Sumption, N. J. Knowles, D. P. King and D. J. Paton	10.1111/j.1865-1682.2010.01151.x
**2010**	Newcastle disease outbreaks in the Sudan from 2003 to 2006 were caused by viruses of genotype 5d	Virus Genes	W. Hassan, S. A. Khair, B. Mochotlhoane and C. Abolnik	10.1007/s11262-009-0424-4
**2010**	Nosocomial outbreak of Crimean-Congo hemorrhagic fever, Sudan	Emerg Infect Dis	I. E. Aradaib, B. R. Erickson, M. E. Mustafa, M. L. Khristova, N. S. Saeed, R. M. Elageb and S. T. Nichol	10.3201/eid1605.091815
**2010**	Ovine clone ST1464: a predominant genotype of Staphylococcus aureus subsp. anaerobius isolated from sheep in Sudan	J Infect Dev Ctries	H. Elbir, E. J. Feil, M. Drancourt, V. Roux, S. M. El Sanousi, M. Eshag, P. Colque-Navarro, I. Kühn and J. I. Flock	10.3855/jidc.632
**2010**	Respiratory infection of camels associated with parainfluenza virus 3 in Sudan	J Virol Methods	K. S. Intisar, Y. H. Ali, A. I. Khalafalla, M. E. Rahman and A. S. Amin	10.1016/j.jviromet.2009.08.017
**2011**	A molecular survey on cystic echinococcosis in Sinnar area, Blue Nile state (Sudan)	Chin Med J (Engl)	K. Ibrahim, R. Thomas, K. Peter and R. A. Omer	PMID: 17519100
**2011**	A nosocomial transmission of crimean-congo hemorrhagic fever to an attending physician in North Kordufan, Sudan	Virol J	A. T. Elata, M. S. Karsany, R. M. Elageb, M. A. Hussain, K. H. Eltom, M. I. Elbashir and I. E. Aradaib	10.1186/1743-422x-8-303

**2011**	Characterization of the complete genomes of Camelus dromedarius papillomavirus types 1 and 2	J Gen Virol	A. E. Ure, A. K. Elfadl, A. I. Khalafalla, A. A. R. Gameel, J. Dillner and O. Forslund	10.1099/vir.0.031039-0
**2011**	Effects of natural selection and gene conversion on the evolution of human glycophorins coding for MNS blood polymorphisms in malaria-endemic African populations	Am J Hum Genet	W. Y. Ko, K. A. Kaercher, E. Giombini, P. Marcatili, A. Froment, M. Ibrahim, G. Lema, T. B. Nyambo, S. A. Omar, C. Wambebe, A. Ranciaro, J. B. Hirbo and S. A. Tishkoff	10.1016/j.ajhg.2011.05.005
**2011**	First Report of Cucurbit chlorotic yellows virus Infecting Muskmelon and Cucumber in Sudan	Plant Dis	K. Hamed, W. Menzel, G. Dafalla, A. M. A. Gadelseed and S. Winter	10.1094/pdis-04-11-0349
**2011**	First Report of Tomato chlorosis virus Infecting Tomato in Sudan	Plant Dis	E. Fiallo-Olivé, A. A. Hamed, E. Moriones and J. Navas-Castillo	10.1094/pdis-08-11-0631
**2011**	Gene expression of monodehydroascorbate reductase and dehydroascorbate reductase during fruit ripening and in response to environmental stresses in acerola (Malpighia glabra)	Journal of Plant Physiology	H. A. Eltelib, A. A. Badejo, Y. Fujikawa and M. Esaka	https://doi.org/10.1016/j.jplph.2010.09.003
**2011**	Genetic characterization of local Sudanese sheep breeds using DNA markers	Small Ruminant Research	N. Gornas, C. Weimann, A. El Hussien and G. Erhardt	https://doi.org/10.1016/j.smallrumres.2010.08.009
**2011**	Hepatitis B virus (HBV) infection and recombination between HBV genotypes D and E in asymptomatic blood donors from Khartoum, Sudan	J Clin Microbiol	S. Mahgoub, D. Candotti, M. El Ekiaby and J. P. Allain	10.1128/jcm.00867-10
**2011**	Identification and safety evaluation of Bacillus species occurring in high numbers during spontaneous fermentations to produce Gergoush, a traditional Sudanese bread snack	International Journal of Food Microbiology	L. Thorsen, W. S. Abdelgadir, M. H. Rønsbo, S. Abban, S. H. Hamad, D. S. Nielsen and M. Jakobsen	https://doi.org/10.1016/j.ijfoodmicro.2011.02.028
**2011**	Microsatellite-Based Genetic Differentiation and Phylogeny of Sheep Breeds in Mongolia Sheep Group of China	Agricultural Sciences in China	W. Sun, H. Chang, H. Musa Hussein, X.-j. Liao, M.-x. Chu and J. Kija	https://doi.org/10.1016/S1671-2927(11)60097-7
**2011**	Multiple Crimean-Congo hemorrhagic fever virus strains are associated with disease outbreaks in Sudan, 2008-2009	PLoS Negl Trop Dis	I. E. Aradaib, B. R. Erickson, M. S. Karsany, M. L. Khristova, R. M. Elageb, M. E. Mohamed and S. T. Nichol	10.1371/journal.pntd.0001159
**2011**	Prevalence and genetic diversity of Babesia and Anaplasma species in cattle in Sudan	Veterinary Parasitology	H. Awad, S. Antunes, R. C. Galindo, V. E. do Rosário, J. de la Fuente, A. Domingos and A. M. El Hussein	https://doi.org/10.1016/j.vetpar.2011.04.007
**2011**	Snake melon asteroid mosaic virus, a Tentative New Member of the Genus Sobemovirus Infecting Cucurbits	Plant Dis	H. Lecoq, G. Dafalla, B. Delécolle, C. Wipf-Scheibel and C. Desbiez	10.1094/pdis-06-10-0447
**2011**	Use of the Capripoxvirus homologue of Vaccinia virus 30kDa RNA polymerase subunit (RPO30) gene as a novel diagnostic and genotyping target: Development of a classical PCR method to differentiate Goat poxvirus from Sheep poxvirus	Veterinary Microbiology	C. E. Lamien, C. Le Goff, R. Silber, D. B. Wallace, V. Gulyaz, E. Tuppurainen, H. Madani, P. Caufour, T. Adam, M. E. Harrak, A. G. Luckins, E. Albina and A. Diallo	https://doi.org/10.1016/j.vetmic.2010.09.038
**2011**	β2-Adrenergic activity of 6-methoxykaempferol-3-O-glucoside on rat uterus: In vitro and in silico studies	European Journal of Pharmacology	A. A. E. Ahmed, A. Marki, R. Gaspar, A. Vasas, M. M. E. Mudawi, J. Verli, B. Jójárt, J. Hohmann and G. Falkay	https://doi.org/10.1016/j.ejphar.2011.05.066

**2012**	Evidence for genetic differentiation at the microgeographic scale in Phlebotomus papatasi populations from Sudan	Parasit Vectors	N. M. Khalid, M. A. Aboud, F. M. Alrabba, D. E. Elnaiem and F. Tripet	10.1186/1756-3305-5-249
**2012**	First Report of Shallot virus X in Onion in Sudan	Plant Dis	K. Hamed, W. Menzel, M. E. Mohamed, G. Dafallah, A. M. A. Gadelseed and S. Winter	10.1094/pdis-03-12-0253-pdn
**2012**	Immunogenicity and immune modulatory effects of in silico predicted L. donovani candidate peptide vaccines	Hum Vaccin Immunother	M. E. Elfaki, E. A. Khalil, A. S. De Groot, A. M. Musa, A. Gutierrez, B. M. Younis, K. A. Salih and A. M. El-Hassan	10.4161/hv.21881
**2012**	In vitro and in vivo anti-inflammatory activities of columbin through the inhibition of cycloxygenase-2 and nitric oxide but not the suppression of NF-κB translocation	European Journal of Pharmacology	S. Ibrahim Abdelwahab, W. Syaed Koko, M. Mohamed Elhassan Taha, S. Mohan, M. Achoui, M. Ameen Abdulla, M. Rais Mustafa, S. Ahmad, M. Ibrahim Noordin, C. Lip Yong, M. Roslan Sulaiman, R. Othman and A. Amir Hassan	https://doi.org/10.1016/j.ejphar.2011.12.024
**2012**	Pleurostomophora ochracea, a novel agent of human eumycetoma with yellow grains	J Clin Microbiol	N. A. Mhmoud, S. A. Ahmed, A. H. Fahal, G. S. de Hoog, A. H. Gerrits van den Ende and W. W. van de Sande	10.1128/jcm.01470-12
**2012**	Quantification of PKC family genes in sporadic breast cancer by qRT-PCR: evidence that PKCι/λ overexpression is an independent prognostic factor	Int J Cancer	K. D. Awadelkarim, C. Callens, C. Rossé, A. Susini, S. Vacher, E. Rouleau, R. Lidereau and I. Bièche	10.1002/ijc.27600
**2013**	Biochemical and molecular characterization of polymorphisms of αs1-casein in Sudanese camel (Camelus dromedarius) milk	International Dairy Journal	E. T. S. Shuiep, I. J. Giambra, I. E. Y. M. El Zubeir and G. Erhardt	https://doi.org/10.1016/j.idairyj.2012.09.002
**2013**	Comparison of the bacterial composition and structure in symptomatic and asymptomatic endodontic infections associated with root-filled teeth using pyrosequencing	PLoS One	A. C. Anderson, A. Al-Ahmad, F. Elamin, D. Jonas, Y. Mirghani, M. Schilhabel, L. Karygianni, E. Hellwig and A. Rehman	10.1371/journal.pone.0084960
**2013**	Current status of equine piroplasmosis in the Sudan	Infect Genet Evol	B. Salim, M. A. Bakheit, J. Kamau and C. Sugimoto	10.1016/j.meegid.2013.02.008
**2013**	Detecting and Characterizing Genomic Signatures of Positive Selection in Global Populations	The American Journal of Human Genetics	X. Liu, Rick T.-H. Ong, Esakimuthu N. Pillai, Abier M. Elzein, Kerrin S. Small, Taane G. Clark, Dominic P. Kwiatkowski and Y.-Y. Teo	https://doi.org/10.1016/j.ajhg.2013.04.021
**2013**	Detection of Caprine-specific Nucleic Acid Sequences in Goat Milk Using Polymerase Chain Reaction	Mater Sociomed	A. A. Osman, I. E. Aradaib and O. A. Musa	10.5455/msm.2013.25.105-108
**2013**	Development and evaluation of loop-mediated isothermal amplification assay for detection of Crimean Congo hemorrhagic fever virus in Sudan	Journal of Virological Methods	H. A. M. Osman, K. H. Eltom, N. O. Musa, N. M. Bilal, M. I. Elbashir and I. E. Aradaib	https://doi.org/10.1016/j.jviromet.2013.03.004
**2013**	Distribution of Class I integrons and their effect on the prevalence of multi-drug resistant Escherichia coli clinical isolates from Sudan	Saudi Med J	M. E. Ibrahim, M. A. Magzoub, N. E. Bilal and M. E. Hamid	PMID: 22040487
**2013**	Distribution of erythrocyte binding antigen 175 (EBA-175) gene dimorphic alleles in Plasmodium falciparum field isolates from Sudan	BMC Infect Dis	A. A. Adam, A. A. Amine, D. A. Hassan, W. H. Omer, B. Y. Nour, A. Z. Jebakumar, M. E. Ibrahim, N. H. Abdulhadi and H. S. Mohamed	10.1186/1471-2334-13-469

**2013**	First Report of Garlic common latent virus Infecting Garlic in Sudan	Plant Dis	K. Hamed, W. Menzel, M. E. Mohamed, K. A. Bakheet and S. Winter	10.1094/pdis-11-12-1018-pdn
**2013**	First report on circulation of Echinococcus ortleppi in the one humped camel (Camelus dromedaries), Sudan	BMC Vet Res	M. E. Ahmed, K. H. Eltom, N. O. Musa, I. A. Ali, F. M. Elamin, M. P. Grobusch and I. E. Aradaib	10.1186/1746-6148-9-127
**2013**	Inhibition of Japanese encephalitis virus infection in vitro and in vivo by pokeweed antiviral protein	Virus Research	H. Z. A. Ishag, C. Li, L. Huang, M.-x. Sun, B. Ni, C.-x. Guo and X. Mao	https://doi.org/10.1016/j.virusres.2012.10.032
**2013**	Molecular characterization and genetic variability at κ-casein gene (CSN3) in camels	Gene	A. Pauciullo, E. S. Shuiep, G. Cosenza, L. Ramunno and G. Erhardt	https://doi.org/10.1016/j.gene.2012.10.083
**2013**	Preliminary screening for water stress tolerance and genetic diversity in wheat (Triticum aestivum L.) cultivars from Sudan	Journal of Genetic Engineering and Biotechnology	M. A. El Siddig, S. Baenziger, I. Dweikat and A. A. El Hussein	https://doi.org/10.1016/j.jgeb.2013.08.004
**2013**	Rift Valley fever, Sudan, 2007 and 2010	Emerg Infect Dis	I. E. Aradaib, B. R. Erickson, R. M. Elageb, M. L. Khristova, S. A. Carroll, I. M. Elkhidir, M. E. Karsany, A. E. Karrar, M. I. Elbashir and S. T. Nichol	10.3201/eid1902.120834
**2013**	Staphylococcus aureus subsp. anaerobius strain ST1464 genome sequence	Stand Genomic Sci	H. Elbir, C. Robert, T. T. Nguyen, G. Gimenez, S. M. El Sanousi, J. I. Flock, D. Raoult and M. Drancourt	10.4056/sigs.3748294
**2014**	A genotypically distinct, melanic variant of Anopheles arabiensis in Sudan is associated with arid environments	Malar J	M. Aboud, A. Makhawi, A. Verardi, F. El Raba'a, D. E. Elnaiem and H. Townson	10.1186/1475-2875-13-492
**2014**	Analysis of ultra-deep pyrosequencing and cloning based sequencing of the basic core promoter/precore/core region of hepatitis B virus using newly developed bioinformatics tools	PLoS One	M. Yousif, T. G. Bell, H. Mudawi, D. Glebe and A. Kramvis	10.1371/journal.pone.0095377
**2014**	Concomitant Infection with Leishmania donovani and L. major in Single Ulcers of Cutaneous Leishmaniasis Patients from Sudan	J Trop Med	A. M. Babiker, S. Ravagnan, A. Fusaro, M. M. Hassan, S. M. Bakheit, M. M. Mukhtar, G. Cattoli and G. Capelli	10.1155/2014/170859
**2014**	Cytotoxicity of the bisphenolic honokiol from Magnolia officinalis against multiple drug-resistant tumor cells as determined by pharmacogenomics and molecular docking	Phytomedicine	M. Saeed, V. Kuete, O. Kadioglu, J. Börtzler, H. Khalid, H. J. Greten and T. Efferth	10.1016/j.phymed.2014.07.011
**2014**	Direct Leishmania species typing in Old World clinical samples: evaluation of 3 sensitive methods based on the heat-shock protein 70 gene	Diagn Microbiol Infect Dis	A. M. Montalvo, J. Fraga, S. El Safi, M. Gramiccia, C. L. Jaffe, J. C. Dujardin and G. Van der Auwera	10.1016/j.diagmicrobio.2014.05.012
**2014**	First Report of Pepper vein yellows virus Infecting Hot Pepper in Sudan	Plant Dis	A. Alfaro-Fernández, E. E. ElShafie, M. A. Ali, O. O. A. El Bashir, M. C. Córdoba-Sellés and M. Ambrosio	10.1094/pdis-03-14-0251-pdn
**2014**	Frequency of mutations in the rpoB gene of multidrug-resistant Mycobacterium tuberculosis clinical isolates from Sudan	J Infect Dev Ctries	H. Elbir and N. Y. Ibrahim	10.3855/jidc.4496
**2014**	Genetic origins of lactase persistence and the spread of pastoralism in Africa	Am J Hum Genet	A. Ranciaro, M. C. Campbell, J. B. Hirbo, W. Y. Ko, A. Froment, P. Anagnostou, M. J. Kotze, M.	10.1016/j.ajhg.2014.02.009

			Ibrahim, T. Nyambo, S. A. Omar and S. A. Tishkoff	
**2014**	Genome-wide identification and expression analysis of the CaNAC family members in chickpea during development, dehydration and ABA treatments	PLoS One	C. V. Ha, M. N. Esfahani, Y. Watanabe, U. T. Tran, S. Sulieman, K. Mochida, D. V. Nguyen and L. S. Tran	10.1371/journal.pone.0114107
**2014**	Genotyping and virological characteristics of hepatitis B virus in HIV-infected individuals in Sudan	Int J Infect Dis	M. Yousif, H. Mudawi, W. Hussein, M. Mukhtar, O. Nemeri, D. Glebe and A. Kramvis	10.1016/j.ijid.2014.07.002
**2014**	Historical demographic profiles and genetic variation of the East African Butana and Kenana indigenous dairy zebu cattle	Anim Genet	B. Salim, K. M. Taha, O. Hanotte and J. M. Mwacharo	10.1111/age.12225
**2014**	Insights into the possible role of IFNG and IFNGR1 in Kala-azar and Post Kala-azar Dermal Leishmaniasis in Sudanese patients	BMC Infect Dis	M. A. Salih, M. Fakiola, M. H. Abdelraheem, B. M. Younis, A. M. Musa, A. M. ElHassan, J. M. Blackwell, M. E. Ibrahim and H. S. Mohamed	10.1186/s12879-014-0662-5
**2014**	Leishmania donovani populations in Eastern Sudan: temporal structuring and a link between human and canine transmission	Parasit Vectors	R. Baleela, M. S. Llewellyn, S. Fitzpatrick, K. Kuhls, G. Schönian, M. A. Miles and I. L. Mauricio	10.1186/s13071-014-0496-4
**2014**	Molecular detection of equine trypanosomes in the Sudan	Vet Parasitol	B. Salim, M. A. Bakheit and C. Sugimoto	10.1016/j.vetpar.2013.09.002
**2014**	Novel NSP1 genotype characterised in an African camel G8P[11] rotavirus strain	Infect Genet Evol	K. C. Jere, M. D. Esona, Y. H. Ali, I. Peenze, S. Roy, M. D. Bowen, I. K. Saeed, A. I. Khalafalla, M. M. Nyaga, J. Mphahlele, D. Steele and M. L. Seheri	10.1016/j.meegid.2013.10.002
**2014**	Rapid identification of black grain eumycetoma causative agents using rolling circle amplification	PLoS Negl Trop Dis	S. A. Ahmed, B. H. van den Ende, A. H. Fahal, W. W. van de Sande and G. S. de Hoog	10.1371/journal.pntd.0003368
**2014**	Roussoella percutanea, a novel opportunistic pathogen causing subcutaneous mycoses	Med Mycol	S. A. Ahmed, D. A. Stevens, W. W. van de Sande, J. F. Meis and G. S. de Hoog	10.1093/mmy/myu035
**2014**	Screening and characterization of RAPD markers in viscerotropic Leishmania parasites	PLoS One	I. Mkada-Driss, R. Lahmadi, A. S. Chakroun, C. Talbi, S. Guerbouj, M. Driss, E. M. Elamine, E. Cupolillo, M. M. Mukhtar and I. Guizani	10.1371/journal.pone.0109773
**2014**	The African baobab (Adansonia digitata, Malvaceae): genetic resources in neglected populations of the Nuba Mountains, Sudan	Am J Bot	M. Wiehle, K. Prinz, K. Kehlenbeck, S. Goenster, S. A. Mohamed, R. Finkeldey, A. Buerkert and J. Gebauer	10.3732/ajb.1400198
**2014**	The episode of genetic drift defining the migration of humans out of Africa is derived from a large east African population size	PLoS One	N. Elhassan, E. I. Gebremeskel, M. A. Elnour, D. Isabirye, J. Okello, A. Hussien, D. Kwiatksowski, J. Hirbo, S. Tishkoff and M. E. Ibrahim	10.1371/journal.pone.0097674
**2014**	The lignan, (−)-sesamin reveals cytotoxicity toward cancer cells: Pharmacogenomic determination of genes associated with sensitivity or resistance	Phytomedicine	M. Saeed, H. Khalid, Y. Sugimoto and T. Efferth	https://doi.org/10.1016/j.phymed.2014.01.006
**2014**	Validation of a recombinant protein indirect ELISA for the detection of specific antibodies against Theileria uilenbergi and Theileria luwenshuni in small ruminants	Veterinary Parasitology	Z. Liu, Y. Li, D. E. A. Salih, J. Luo, J. S. Ahmed, U. Seitzer and H. Yin	https://doi.org/10.1016/j.vetpar.2014.05.010

**2014**	Y-chromosome E haplogroups: their distribution and implication to the origin of Afro-Asiatic languages and pastoralism	Eur J Hum Genet	E. I. Gebremeskel and M. E. Ibrahim	10.1038/ejhg.2014.41
**2015**	Activity of the dietary flavonoid, apigenin, against multidrug-resistant tumor cells as determined by pharmacogenomics and molecular docking	J Nutr Biochem	M. Saeed, O. Kadioglu, H. Khalid, Y. Sugimoto and T. Efferth	10.1016/j.jnutbio.2014.09.008
**2015**	Alternatively spliced transcripts and novel pseudogenes of the Plasmodium falciparum resistance-associated locus pfcrt detected in East African malaria patients	J Antimicrob Chemother	N. B. Gadalla, M. Malmberg, I. Adam, M. C. Oguike, K. Beshir, S. E. Elzaki, I. Mukhtar, A. A. Gadalla, D. C. Warhurst, B. Ngasala, A. Mårtensson, B. B. El-Sayed, J. P. Gil and C. J. Sutherland	10.1093/jac/dku358
**2015**	Candidate gene analysis supports a role for polymorphisms at TCF7L2 as risk factors for type 2 diabetes in Sudan	J Diabetes Metab Disord	A. T. Ibrahim, A. Hussain, M. A. Salih, O. A. Ibrahim, S. E. Jamieson, M. E. Ibrahim, J. M. Blackwell and H. S. Mohamed	10.1186/s40200-016-0225-y
**2015**	COI gene sequence analysis for testing cyclical mating in securing genetic diversity of Macrobrachium rosenbergii	Biochemical Systematics and Ecology	M. O. Elsheikh, F. Begham Mustafa, I. Ibrahim Eid, A. Lutas and S. Bhassu	https://doi.org/10.1016/j.bse.2015.07.040
**2015**	Cytotoxicity of 35 medicinal plants from Sudan towards sensitive and multidrug-resistant cancer cells	Journal of Ethnopharmacology	M. E. M. Saeed, H. Abdelgadir, Y. Sugimoto, H. E. Khalid and T. Efferth	https://doi.org/10.1016/j.jep.2015.07.005
**2015**	Cytotoxicity of the Sesquiterpene Lactones Neoambrosin and Damsin from Ambrosia maritima Against Multidrug-Resistant Cancer Cells	Front Pharmacol	M. Saeed, S. Jacob, L. P. Sandjo, Y. Sugimoto, H. E. Khalid, T. Opatz, E. Thines and T. Efferth	10.3389/fphar.2015.00267
**2015**	DT2008: a promising new genetic resource for improved drought tolerance in soybean when solely dependent on symbiotic N2 fixation	Biomed Res Int	S. Sulieman, C. Van Ha, M. Nasr Esfahani, Y. Watanabe, R. Nishiyama, C. T. Pham, D. Van Nguyen and L. S. Tran	10.1155/2015/687213
**2015**	Evaluation of spa-typing of methicillin-resistant Staphylococcus aureus using high-resolution melting analysis	Int J Infect Dis	W. Mazi, V. Sangal, G. Sandstrom, A. Saeed and J. Yu	10.1016/j.ijid.2015.05.002
**2015**	Exome sequencing of a colorectal cancer family reveals shared mutation pattern and predisposition circuitry along tumor pathways	Front Genet	S. H. Suleiman, M. E. Koko, W. H. Nasir, O. Elfateh, U. K. Elgizouli, M. O. Abdallah, K. O. Alfarouk, A. Hussain, S. Faisal, F. M. Ibrahim, M. Romano, A. Sultan, L. Banks, M. Newport, F. Baralle, A. M. Elhassan, H. S. Mohamed and M. E. Ibrahim	10.3389/fgene.2015.00288
**2015**	Genetic determinants of anti-malarial acquired immunity in a large multi-centre study	Malar J	J. M. Shelton, P. Corran, P. Risley, N. Silva, C. Hubbart, A. Jeffreys, K. Rowlands, R. Craik, V. Cornelius, M. Hensmann, S. Molloy, N. Sepulveda, T. G. Clark, G. Band, G. M. Clarke, C. C. Spencer, A. Kerasidou, S. Campino, S. Auburn, A. Tall, A. B. Ly, O. Mercereau-Puijalon, A. Sakuntabhai, A. Djimdé, B. Maiga, O. Touré, O. K. Doumbo, A. Dolo, M. Troye-Blomberg, V. D. Mangano, F.	10.1186/s12936-015-0833-x

			Verra, D. Modiano, E. Bougouma, S. B. Sirima, M. Ibrahim, A. Hussain, N. Eid, A. Elzein, H. Mohammed, A. Elhassan, I. Elhassan, T. N. Williams, C. Ndila, A. Macharia, K. Marsh, A. Manjurano, H. Reyburn, M. Lemnge, D. Ishengoma, R. Carter, N. Karunaweera, D. Fernando, R. Dewasurendra, C. J. Drakeley, E. M. Riley, D. P. Kwiatkowski and K. A. Rockett	
**2015**	Genetic Diversity of Schistosoma haematobium Eggs Isolated from Human Urine in Sudan	Korean J Parasitol	J. H. Quan, I. W. Choi, H. A. Ismail, A. S. Mohamed, H. G. Jeong, J. S. Lee, S. T. Hong, T. S. Yong, G. H. Cha and Y. H. Lee	10.3347/kjp.2015.53.3.271
**2015**	In vivo formation of Plasmodium falciparum ribosomal stalk - a unique mode of assembly without stable heterodimeric intermediates	Biochim Biophys Acta	L. Wawiórka, D. Krokowski, Y. Gordiyenko, D. Krowarsch, C. V. Robinson, I. Adam, N. Grankowski and M. Tchórzewski	10.1016/j.bbagen.2014.10.015
**2015**	In-silico identification of the binding mode of synthesized adamantyl derivatives inside cholinesterase enzymes	Acta Pharmacol Sin	A. Al-Aboudi, R. A. Al-Qawasmeh, A. Shahwan, U. Mahmood, A. Khalid and Z. Ul-Haq	10.1038/aps.2014.173
**2015**	Molecular diversity of Chickpea chlorotic dwarf virus in Sudan: high rates of intra-species recombination - a driving force in the emergence of new strains	Infect Genet Evol	S. Kraberger, S. G. Kumari, A. A. Hamed, B. Gronenborn, J. E. Thomas, M. Sharman, G. W. Harkins, B. M. Muhire, D. P. Martin and A. Varsani	10.1016/j.meegid.2014.11.024
**2015**	Molecular genetic analysis of Plasmodium vivax isolates from Eastern and Central Sudan using pvcsp and pvmsp-3α genes as molecular markers	Infection, Genetics and Evolution	A. A. Talha, S. Pirahmadi, A. A. Mehrizi, N. D. Djadid, B. Y. M. Nour and S. Zakeri	https://doi.org/10.1016/j.meegid.2015.02.004
**2015**	Multiplex PCR for rapid diagnosis and differentiation of pox and pox-like diseases in dromedary Camels	Virol J	A. I. Khalafalla, K. A. Al-Busada and I. M. El-Sabagh	10.1186/s12985-015-0329-x
**2015**	Phaeohyphomycosis Caused by a Novel Species, Pseudochaetosphaeronema martinelli	J Clin Microbiol	S. A. Ahmed, N. Desbois, D. Quist, C. Miossec, C. Atoche, A. Bonifaz and G. S. de Hoog	10.1128/jcm.01456-15
**2015**	Pharmacogenomic and molecular docking studies on the cytotoxicity of the natural steroid wortmannin against multidrug-resistant tumor cells	Phytomedicine	V. Kuete, M. E. M. Saeed, O. Kadioglu, J. Börtzler, H. Khalid, H. J. Greten and T. Efferth	https://doi.org/10.1016/j.phymed.2014.11.011
**2015**	Phylogenetic analysis of eight sudanese camel contagious ecthyma viruses based on B2L gene sequence	Virol J	A. I. Khalafalla, I. M. El-Sabagh, K. A. Al-Busada, A. I. Al-Mubarak and Y. H. Ali	10.1186/s12985-015-0348-7
**2015**	Plasmodium falciparum population structure in Sudan post artemisinin-based combination therapy	Acta Trop	A. M. Bakhiet, A. M. Abdel-Muhsin, S. E. Elzaki, Z. Al-Hashami, H. S. Albarwani, B. A. AlQamashoui, S. Al-Hamidhi, M. A. Idris, A. A. Elagib, A. Beja-Pereira and H. A. Babiker	10.1016/j.actatropica.2015.04.013
**2015**	Plasmodium vivax Diversity and Population Structure across Four Continents	PLoS Negl Trop Dis	C. Koepfli, P. T. Rodrigues, T. Antao, P. Orjuela-Sánchez, P. Van den Eede, D. Gamboa, N. van Hong, J. Bendezu, A. Erhart, C. Barnadas, A.	10.1371/journal.pntd.0003872

			Ratsimbasoa, D. Menard, C. Severini, M. Menegon, B. Y. Nour, N. Karunaweera, I. Mueller, M. U. Ferreira and I. Felger	
**2015**	Prevalence of protozoa species in drinking and environmental water sources in Sudan	Biomed Res Int	S. Shanan, H. Abd, M. Bayoumi, A. Saeed and G. Sandström	10.1155/2015/345619
**2015**	Proteomic analysis of homocholine catabolic pathway in Pseudomonas sp. strain A9	Process Biochemistry	I. A. Mohamed Ahmed, M. E. Eltayeb, N. Mori, J. Arima, H. Tanaka, T. Taniguchi and N. Yamanaka	https://doi.org/10.1016/j.procbio.2015.07.001
**2015**	Rare zoonotic infection with Microsporum persicolor with literature review	Mycoses	P. Krzyściak, A. M. Al-Hatmi, S. A. Ahmed and A. B. Macura	10.1111/myc.12341
**2015**	Replication of the association of GLT6D1 with aggressive periodontitis in a Sudanese population	J Clin Periodontol	N. T. Hashim, G. J. Linden, M. E. Ibrahim, B. G. Gismalla, F. T. Lundy, F. J. Hughes and I. A. El Karim	10.1111/jcpe.12375
**2015**	Spatiotemporal dynamics of Puumala hantavirus associated with its rodent host, Myodes glareolus	Evol Appl	V. Weber de Melo, H. Sheikh Ali, J. Freise, D. Kühnert, S. Essbauer, M. Mertens, K. M. Wanka, S. Drewes, R. G. Ulrich and G. Heckel	10.1111/eva.12263
**2015**	Supramolecular interaction of gemifloxacin and hydroxyl propyl β-cyclodextrin spectroscopic characterization, molecular modeling and analytical application	Spectrochim Acta A Mol Biomol Spectrosc	N. F. Dsugi, A. A. Elbashir and F. E. Suliman	10.1016/j.saa.2015.06.031
**2015**	Tuberculosis drug resistance isolates from pulmonary tuberculosis patients, Kassala State, Sudan	Int J Mycobacteriol	F. A. Khalid, Z. A. Hamid and M. M. Mukhtar	10.1016/j.ijmyco.2014.11.064
**2015**	Valproic acid as a potential inhibitor of Plasmodium falciparum histone deacetylase 1 (PfHDAC1): an in silico approach	Int J Mol Sci	M. A. Elbadawi, M. K. Awadalla, M. M. Hamid, M. A. Mohamed and T. A. Awad	10.3390/ijms16023915
**2016**	A replicating plasmid-based vector for GFP expression in Mycoplasma hyopneumoniae	Genet Mol Res	H. Z. Ishag, M. J. Liu, R. S. Yang, Q. Y. Xiong, Z. X. Feng and G. Q. Shao	10.4238/gmr.15027832
**2016**	A Single Lineage of Hepatitis E Virus Causes Both Outbreaks a nd Sporadic Hepatitis in Sudan	Viruses	A. H. Elduma, M. M. Zein, M. Karlsson, I. M. Elkhidir and H. Norder	10.3390/v8100273
**2016**	Adapting Document Similarity Measures for Ligand-Based Virtual Screening	Molecules	M. Himmat, N. Salim, M. M. Al-Dabbagh, F. Saeed and A. Ahmed	10.3390/molecules21040476
**2016**	Alpha S1-casein polymorphisms in camel (Camelus dromedarius) and descriptions of biological active peptides and allergenic epitopes	Trop Anim Health Prod	G. Erhardt, T. S. Shuiep el, M. Lisson, C. Weimann, Z. Wang, Y. El Zubeir Iel and A. Pauciullo	10.1007/s11250-016-0997-6
**2016**	Alteration of wheat vernalization requirement by alien chromosome-mediated transposition of MITE	Breed Sci	Y. S. Gorafi, A. E. Eltayeb and H. Tsujimoto	10.1270/jsbbs.66.181
**2016**	Ancient and modern DNA reveal dynamics of domestication and cross-continental dispersal of the dromedary	Proc Natl Acad Sci U S A	F. Almathen, P. Charruau, E. Mohandesan, J. M. Mwacharo, P. Orozco-terWengel, D. Pitt, A. M. Abdussamad, M. Uerpmann, H. P. Uerpmann, B. De Cupere, P. Magee, M. A. Alnaqeeb, B. Salim, A. Raziq, T. Dessie, O. M. Abdelhadi, M. H.	10.1073/pnas.1519508113

			Banabazi, M. Al-Eknah, C. Walzer, B. Faye, M. Hofreiter, J. Peters, O. Hanotte and P. A. Burger	
**2016**	Association analysis of IGF-I gene expression with growth and reproductive traits in Jinghai yellow chickens	Genet Mol Res	M. A. Abdalhag, T. Li, L. Duan, T. Zhang, G. Zhang, J. Wang and Y. Wang	10.4238/gmr15049205
**2016**	Association of IL-37 gene polymorphisms with susceptibility to tuberculosis in Saudi subjects	Microbiol Immunol	G. Allam, I. A. Mohamed, K. A. Alswat, S. H. Abbadi, R. Nassif, B. J. Alharthi and A. Nasr	10.1111/1348-0421.12444
**2016**	Association of Rift Valley fever virus infection with miscarriage in Sudanese women: a cross-sectional study	The Lancet Global Health	M. Baudin, A. M. Jumaa, H. J. E. Jomma, M. S. Karsany, G. Bucht, J. Näslund, C. Ahlm, M. Evander and N. Mohamed	https://doi.org/10.1016/S2214-109X(16)30176-0
**2016**	Bioinformatics Approach for Prediction of Functional Coding/Noncoding Simple Polymorphisms (SNPs/Indels) in Human BRAF Gene	Adv Bioinformatics	M. M. Hassan, S. E. Omer, R. M. Khalf-Allah, R. Y. Mustafa, I. S. Ali and S. B. Mohamed	10.1155/2016/2632917
**2016**	Candidate genes for the development of hair follicles in Hu sheep	Genet Mol Res	X. Y. Lv, R. Ni, W. Sun, R. Su, H. H. Musa, J. F. Yin, Q. Z. Wang, W. Gao and L. Chen	10.4238/gmr.15036877
**2016**	Characterization and occurrence of squash chlorotic leaf spot virus, a tentative new torradovirus infecting cucurbits in Sudan	Arch Virol	H. Lecoq, E. Verdin, M. Tepfer, C. Wipf-Scheibel, P. Millot, G. Dafalla and C. Desbiez	10.1007/s00705-016-2797-8
**2016**	Characterization of a new cucurbit-infecting ipomovirus from Sudan	Arch Virol	C. Desbiez, E. Verdin, M. Tepfer, C. Wipf-Scheibel, P. Millot, G. Dafalla and H. Lecoq	10.1007/s00705-016-2981-x
**2016**	Comparative sequence analysis of domain I of Plasmodium falciparum apical membrane antigen 1 from Saudi Arabia and worldwide isolates	Infect Genet Evol	A. A. Al-Qahtani, A. A. Abdel-Muhsin, S. M. B. Dajem, A. A. H. AlSheikh, M. F. F. Bohol, M. N. Al-Ahdal, C. Putaporntip and S. Jongwutiwes	10.1016/j.meegid.2016.02.010
**2016**	Distribution of coat-color-associated alleles in the domestic horse population and Przewalski's horse	J Appl Genet	M. Reissmann, L. Musa, S. Zakizadeh and A. Ludwig	10.1007/s13353-016-0352-7
**2016**	Endophytic fungi associated with Sudanese medicinal plants show cytotoxic and antibiotic potential	FEMS Microbiol Lett	A. Khiralla, I. E. Mohamed, T. Tzanova, H. Schohn, S. Slezack-Deschaumes, A. Hehn, P. André, G. Carre, R. Spina, A. Lobstein, S. Yagi and D. Laurain-Mattar	10.1093/femsle/fnw089
**2016**	Evaluating ancient Egyptian prescriptions today: Anti-inflammatory activity of Ziziphus spina-christi	Phytomedicine	O. Kadioglu, S. Jacob, S. Bohnert, J. Naß, M. E. M. Saeed, H. Khalid, I. Merfort, E. Thines, T. Pommerening and T. Efferth	https://doi.org/10.1016/j.phymed.2016.01.004
**2016**	Evaluation of in vitro and in vivo anti-inflammatory effects of (-)-pseudosemiglabrin, a major phytoconstituent isolated from Tephrosia apollinea (Delile) DC	J Ethnopharmacol	L. E. Hassan, S. S. Dahham, S. M. Fadul, M. I. Umar, A. S. Majid, K. Y. Khaw and A. M. Majid	10.1016/j.jep.2016.08.023
**2016**	Evidence of an Exponential Decay Pattern of the Hepatitis Delta Virus Evolution Rate and Fluctuations in Quasispecies Complexity in Long-Term Studies of Chronic Delta Infection	PLoS One	M. Homs, F. Rodriguez-Frias, J. Gregori, A. Ruiz, P. Reimundo, R. Casillas, D. Tabernero, C. Godoy, S. Barakat, J. Quer, M. Riveiro-Barciela, M. Roggendorf, R. Esteban and M. Buti	10.1371/journal.pone.0158557

**2016**	Expression of mitochondria-related genes is elevated in overfeeding-induced goose fatty liver	Comp Biochem Physiol B Biochem Mol Biol	R. H. Osman, D. Shao, L. Liu, L. Xia, X. Sun, Y. Zheng, L. Wang, R. Zhang, Y. Zhang, J. Zhang, D. Gong and T. Geng	10.1016/j.cbpb.2015.11.006
**2016**	Full genome sequence analysis of a newly emerged QX-like infectious bronchitis virus from Sudan reveals distinct spots of recombination	Infect Genet Evol	M. M. Naguib, D. Höper, A. S. Arafa, A. M. Setta, M. Abed, I. Monne, M. Beer and T. C. Harder	10.1016/j.meegid.2016.10.017
**2016**	Genome Sequence of Madurella mycetomatis mm55, Isolated from a Human Mycetoma Case in Sudan	Genome Announc	S. Smit, M. F. Derks, S. Bervoets, A. Fahal, W. van Leeuwen, A. van Belkum and W. W. van de Sande	10.1128/genomeA.00418-16
**2016**	Genome-wide characterization and expression analysis of MYB transcription factors in Gossypium hirsutum	BMC Genet	H. Salih, W. Gong, S. He, G. Sun, J. Sun and X. Du	10.1186/s12863-016-0436-8
**2016**	Griffithsin binds to the glycosylated proteins (E and prM) of Japanese encephalitis virus and inhibit its infection	Virus Research	H. Z. A. Ishag, C. Li, F. Wang and X. Mao	https://doi.org/10.1016/j.virusres.2016.01.016
**2016**	H3ABioNet, a sustainable pan-African bioinformatics network for human heredity and health in Africa	Genome Res	N. J. Mulder, E. Adebiyi, R. Alami, A. Benkahla, J. Brandful, S. Doumbia, D. Everett, F. M. Fadlelmola, F. Gaboun, S. Gaseitsiwe, H. Ghazal, S. Hazelhurst, W. Hide, A. Ibrahimi, Y. Jaufeerally Fakim, C. V. Jongeneel, F. Joubert, S. Kassim, J. Kayondo, J. Kumuthini, S. Lyantagaye, J. Makani, A. Mansour Alzohairy, D. Masiga, A. Moussa, O. Nash, O. Ouwe Missi Oukem-Boyer, E. Owusu-Dabo, S. Panji, H. Patterton, F. Radouani, K. Sadki, F. Seghrouchni, Ö. Tastan Bishop, N. Tiffin and N. Ulenga	10.1101/gr.196295.115
**2016**	Hereditary spastic paraplegias: identification of a novel SPG57 variant affecting TFG oligomerization and description of HSP subtypes in Sudan	Eur J Hum Genet	L. E. Elsayed, I. N. Mohammed, A. A. Hamed, M. A. Elseed, A. Johnson, M. Mairey, H. E. Mohamed, M. N. Idris, M. A. Salih, S. M. El-Sadig, M. E. Koko, A. Y. Mohamed, L. Raymond, M. Coutelier, F. Darios, R. A. Siddig, A. K. Ahmed, A. M. Babai, H. M. Malik, Z. M. Omer, E. O. Mohamed, H. B. Eltahir, N. A. Magboul, E. E. Bushara, A. Elnour, S. M. Rahim, A. Alattaya, M. I. Elbashir, M. E. Ibrahim, A. Durr, A. Audhya, A. Brice, A. E. Ahmed and G. Stevanin	10.1038/ejhg.2016.108
**2016**	Identification and characterization of microRNAs expressed in the African malaria vector Anopheles funestus life stages using high throughput sequencing	Malar J	M. Allam, B. L. Spillings, H. Abdalla, D. Mapiye, L. L. Koekemoer and A. Christoffels	10.1186/s12936-016-1591-0
**2016**	In Silico Analysis of SNPs in PARK2 and PINK1 Genes That Potentially Cause Autosomal Recessive Parkinson Disease	Adv Bioinformatics	Y. H. Bakhit, M. O. Ibrahim, M. Amin, Y. A. Mirghani and M. A. Hassan	10.1155/2016/9313746

**2016**	In vitro protective efficacy of Lithium chloride against Mycoplasma hyopneumoniae infection	Res Vet Sci	H. Z. Ishag, Y. Z. Wu, M. J. Liu, Q. Y. Xiong, Z. X. Feng, R. S. Yang and G. Q. Shao	10.1016/j.rvsc.2016.03.013
**2016**	Laboratory rearing of Anopheles arabiensis: impact on genetic variability and implications for Sterile Insect Technique (SIT) based mosquito control in northern Sudan	Malar J	R. S. Azrag, K. Ibrahim, C. Malcolm, E. E. Rayah and B. El-Sayed	10.1186/s12936-016-1484-2
**2016**	Link of a ubiquitous human coronavirus to dromedary camels	Proc Natl Acad Sci U S A	V. M. Corman, I. Eckerle, Z. A. Memish, A. M. Liljander, R. Dijkman, H. Jonsdottir, K. J. Juma Ngeiywa, E. Kamau, M. Younan, M. Al Masri, A. Assiri, I. Gluecks, B. E. Musa, B. Meyer, M. A. Müller, M. Hilali, S. Bornstein, U. Wernery, V. Thiel, J. Jores, J. F. Drexler and C. Drosten	10.1073/pnas.1604472113
**2016**	MicroRNA expression profiles in response to drought stress in Sorghum bicolor	Gene Expression Patterns	N. B. Hamza, N. Sharma, A. Tripathi and N. Sanan-Mishra	https://doi.org/10.1016/j.gep.2016.01.001
**2016**	Molecular cloning and expression analysis of Fem1b from oriental river prawn Macrobrachium nipponense	Genet Mol Res	N. M. Rahman, H. Fu, H. Qiao, S. Jin, H. Bai, W. Zhang, F. W. Jiang, G. Liang, S. Sun, Y. Gong, F. F. Jiang, Y. Xiong and Y. Wu	10.4238/gmr.15027950
**2016**	Molecular cloning and expression pattern of oriental river prawn (Macrobrachium nipponense) nitric oxide synthase	Genet Mol Res	N. M. Rahman, H. T. Fu, S. M. Sun, H. Qiao, S. Jin, H. K. Bai, W. Y. Zhang, G. X. Liang, Y. S. Gong, Y. W. Xiong and Y. Wu	10.4238/gmr.15038541
**2016**	Molecular cloning, polymorphism, and functional activity of the bovine and water buffalo Mx2 gene promoter region	Springerplus	H. A. Babiker, T. Saito, Y. Nakatsu, S. Takasuga, M. Morita, Y. Sugimoto, J. Ueda and T. Watanabe	10.1186/s40064-016-3729-5
**2016**	Paediatric and adult soft tissue sarcomas with NTRK1 gene fusions: a subset of spindle cell sarcomas unified by a prominent myopericytic/haemangiopericytic pattern	J Pathol	F. Haller, J. Knopf, A. Ackermann, M. Bieg, K. Kleinheinz, M. Schlesner, E. A. Moskalev, R. Will, A. A. Satir, I. E. Abdelmagid, J. Giedl, R. Carbon, O. Rompel, A. Hartmann, S. Wiemann, M. Metzler and A. Agaimy	10.1002/path.4701
**2016**	Phylogenetic analysis of some Newcastle disease virus isolates from the Sudan	Open Vet J	N. A. Elmardi, M. A. Bakheit and A. I. Khalafalla	10.4314/ovj.v6i2.4
**2016**	Population genetic analysis of Theileria parva isolated in cattle and buffaloes in Tanzania using minisatellite and microsatellite markers	Vet Parasitol	E. Rukambile, E. Machuka, M. Njahira, M. Kyalo, R. Skilton, E. Mwega, A. Chota, M. Mathias, R. Sallu and D. Salih	10.1016/j.vetpar.2016.04.038
**2016**	Pulmonary nocardiosis caused by Nocardia otitidiscaviarum in an adult asthmatic female patient: The presence of acid-fast branching filaments is always significant	S Afr Med J	A. Mahgoub, S. A. Gumaa, M. R. Joseph, M. S. Saleh, A. H. Elsheikh, A. I. Elkhalifa, E. Elhaj, R. R. Salih and M. E. Hamid	10.7196/SAMJ.2016.v107.i1.12006
**2017**	Association of NOS3 gene polymorphisms with essential hypertension in Sudanese patients: a case control study	BMC Med Genet	S. Gamil, J. Erdmann, I. B. Abdalrahman and A. O. Mohamed	10.1186/s12881-017-0491-7
**2017**	Association of single nucleotide polymorphism in melatonin receptor 1A gene with egg production traits in Yangzhou geese	Anim Genet	M. A. Alsiddig, S. G. Yu, Z. X. Pan, H. Widaa, T. M. Badri, J. Chen and H. L. Liu	10.1111/age.12517

**2017**	Biophysical and In Silico Studies of the Interaction between the Anti-Viral Agents Acyclovir and Penciclovir, and Human Serum Albumin	Molecules	A. S. Abdelhameed, A. H. Bakheit, F. M. Almutairi, H. AlRabiah and A. A. Kadi	10.3390/molecules22111906
**2017**	Designing a course model for distance-based online bioinformatics training in Africa: The H3ABioNet experience	PLoS Comput Biol	K. T. Gurwitz, S. Aron, S. Panji, S. Maslamoney, P. L. Fernandes, D. P. Judge, A. Ghouila, J. B. Domelevo Entfellner, F. Z. Guerfali, C. Saunders, A. Mansour Alzohairy, S. P. Salifu, R. Ahmed, R. Cloete, J. Kayondo, D. Ssemwanga and N. Mulder	10.1371/journal.pcbi.1005715
**2017**	Detection and sequencing of rotavirus among sudanese children	Pan Afr Med J	M. A. Magzoub, N. E. Bilal, J. A. Bilal, M. A. Alzohairy, B. K. Elamin and G. I. Gasim	10.11604/pamj.2017.28.87.11008
**2017**	Development of oriC-plasmids for use in Mycoplasma hyorhinis	Sci Rep	H. Z. A. Ishag, Q. Xiong, M. Liu, Z. Feng and G. Shao	10.1038/s41598-017-10519-3
**2017**	Discovery and biochemical characterization of a mannose phosphorylase catalyzing the synthesis of novel β-1,3-mannosides	Biochimica et Biophysica Acta (BBA) - General Subjects	F. N. Awad, P. Laborda, M. Wang, A. M. Lu, Q. Li, Z. P. Cai, L. Liu and J. Voglmeir	https://doi.org/10.1016/j.bbagen.2017.09.013
**2017**	Distribution and variability of deformed wing virus of honeybees (Apis mellifera) in the Middle East and North Africa	Insect Sci	N. J. Haddad, A. Noureddine, B. Al-Shagour, W. Loucif-Ayad, M. A. El-Niweiri, E. Anaswah, W. A. Hammour, D. El-Obeid, A. Imad, M. A. Shebl, A. S. Almaleky, A. Nasher, N. Walid, M. F. Bergigui, O. Yañez and J. R. de Miranda	10.1111/1744-7917.12277
**2017**	Draft Genome Sequence of a Multidrug-Resistant Pseudomonas aeruginosa Strain Isolated from a Patient with a Urinary Tract Infection in Khartoum, Sudan	Genome Announc	M. Hussain, M. Suliman, A. Ahmed, H. Altayb and E. Elneima	10.1128/genomeA.00203-17
**2017**	Exome Sequencing Identifies Two Variants of the Alkylglycerol Monooxygenase Gene as a Cause of Relapses in Visceral Leishmaniasis in Children, in Sudan	J Infect Dis	S. Marquet, B. Bucheton, C. Reymond, L. Argiro, S. H. El-Safi, M. M. Kheir, J. P. Desvignes, C. Béroud, A. Mergani, A. Hammad and A. J. Dessein	10.1093/infdis/jix277
**2017**	First Complete Genome Sequence of Methicillin-Resistant Staphylococcus aureus Strain SO-1977 Isolated from Khartoum, Sudan	Genome Announc	S. B. Mohamed, M. S. Ali, F. M. Alamir, T. B. Alyas, A. E. Ahmed, A. O. Seed and R. A. Omer	10.1128/genomeA.00945-17
**2017**	Frequency of c.35delG Mutation in GJB2 Gene (Connexin 26) in Syrian Patients with Nonsyndromic Hearing Impairment	Genet Res Int	H. Kaheel, A. Breß, M. A. Hassan, A. A. Shah, M. Amin, Y. H. Y. Bakhit and M. Kniper	10.1155/2017/5836525
**2017**	Functional role of ompF and ompC porins in pathogenesis of avian pathogenic Escherichia coli	Microb Pathog	H. M. A. Hejair, Y. Zhu, J. Ma, Y. Zhang, Z. Pan, W. Zhang and H. Yao	10.1016/j.micpath.2017.02.033
**2017**	Genes encoding two Theileria parva antigens recognized by CD8+ T-cells exhibit sequence diversity in South Sudanese cattle populations but the majority of alleles are similar to the Muguga component of the live vaccine cocktail	PLoS One	D. A. Salih, R. Pelle, J. M. Mwacharo, M. N. Njahira, W. L. Marcellino, H. Kiara, A. K. Malak, A. R. El Hussein, R. Bishop and R. A. Skilton	10.1371/journal.pone.0171426
**2017**	Genetic differentiation of the G6/7 cluster of Echinococcus canadensis based on mitochondrial marker genes	Int J Parasitol	F. Addy, M. Wassermann, D. Kagendo, D. Ebi, E. Zeyhle, I. E. Elmahdi, G. Umhang, A. Casulli, M.	10.1016/j.ijpara.2017.06.003

			F. Harandi, O. Aschenborn, P. Kern, U. Mackenstedt and T. Romig	
**2017**	Genetic diversity of Plasmodium vivax metacaspase 1 and Plasmodium vivax multi-drug resistance 1 genes of field isolates from Mauritania, Sudan and Oman	Malar J	F. Sow, G. Bonnot, B. R. Ahmed, S. M. Diagana, H. Kebe, M. Koita, B. M. Samba, S. K. Al-Mukhaini, M. Al-Zadjali, S. S. Al-Abri, O. A. Ali, A. M. Samy, M. M. Hamid, M. M. Ali Albsheer, B. Simon, A. L. Bienvenu, E. Petersen and S. Picot	10.1186/s12936-017-1687-1
**2017**	Genetic polymorphism and population structure of Echinococcus ortleppi	Parasitology	F. Addy, M. Wassermann, F. Banda, H. Mbaya, J. Aschenborn, O. Aschenborn, P. Koskei, G. Umhang, D. L. R. M, I. E. Elmahdi, U. Mackenstedt, P. Kern and T. Romig	10.1017/s0031182016001840
**2017**	Genetic Structure of the Western and Eastern African Sahel/Savannah Belt and the Role of Nomadic Pastoralists as Inferred from the Variation of D-Loop Mitochondrial DNA Sequences	Hum Biol	M. Čížková, P. Munclinger, M. Y. Diallo, I. Kulichová, M. G. Mokhtar, A. Dème, L. Pereira and V. Černý	PMID: 30047317
**2017**	Genetic Tracing of Jatropha curcas L. from Its Mesoamerican Origin to the World	Front Plant Sci	H. Li, S. Tsuchimoto, K. Harada, M. Yamasaki, H. Sakai, N. Wada, A. Alipour, T. Sasai, A. Tsunekawa, H. Tsujimoto, T. Ando, H. Tomemori, S. Sato, H. Hirakawa, V. P. Quintero, A. Zamarripa, P. Santos, A. Hegazy, A. M. Ali and K. Fukui	10.3389/fpls.2017.01539
**2017**	Genotyping of Theileria lestoquardi from sheep and goats in Sudan to support control of Malignant Ovine Theileriosis	Vet Parasitol	A. M. Ali, D. A. Salih, M. N. Njahira, S. K. Hassan, A. M. El Hussein, Z. Liu, H. Yin, R. Pelle and R. A. Skilton	10.1016/j.vetpar.2017.04.005
**2017**	High altitude and pre-eclampsia: Adaptation or protection	Med Hypotheses	S. I. Y. Ahmed, M. E. Ibrahim and E. A. G. Khalil	10.1016/j.mehy.2017.05.007
**2017**	Human and Dromedary Camel Infection with Camelpox Virus in Eastern Sudan	Vector Borne Zoonotic Dis	A. I. Khalafalla and F. Abdelazim	10.1089/vbz.2016.2070
**2017**	Identification and Characterization of the Diverse Stress-Responsive R2R3-RMYB Transcription Factor from Hibiscus sabdariffa L	Int J Genomics	B. B. Mohamed, B. Aftab, M. B. Sarwar, B. Rashid, Z. Ahmad, S. Hassan and T. Husnain	10.1155/2017/2763259
**2017**	Identification of a novel linear B-cell epitope in the p27 of Avian leukosis virus	Virus Res	W. O. A. Khairy, L. Wang, X. Tian, J. Ye, K. Qian, H. Shao and A. Qin	10.1016/j.virusres.2017.07.007
**2017**	Identification of two conserved B-cell epitopes in the gp90 of reticuloendothelial virus using peptide microarray	Vet Microbiol	W. O. A. Khairy, K. Qian, H. Shao, J. Ye and A. Qin	10.1016/j.vetmic.2017.10.009
**2017**	In silico analysis of single nucleotide polymorphisms (SNPs) in human FOXC2 gene	F1000Res	M. Nimir, M. Abdelrahim, M. Abdelrahim, M. Abdalla, W. E. Ahmed, M. Abdullah and M. M. A. Hamid	10.12688/f1000research.10937.2
**2017**	Investigation on papillomavirus infection in dromedary camels in Al-Ahsa, Saudi Arabia	Open Vet J	A. I. Khalafalla, R. O. Ramadan, A. Rector and S. Barakat	10.4314/ovj.v7i2.16

**2017**	Isolation and partial characterization of 3 nontoxic d-galactose-specific isolectins from seeds of Momordica balsamina	J Mol Recognit	A. K. Awadallah, M. E. Osman, M. A. Ibrahim, E. S. Bernardes, M. Dias-Baruffi and E. H. Konozy	10.1002/jmr.2582
**2017**	Maternal genetic diversity and phylogeography of native Arabian goats	Livestock Science	N. A. Al-Araimi, R. M. Al-Atiyat, O. M. Gaafar, R. Vasconcelos, A. Luzuriaga-Neira, M. O. Eisa, N. Amir, M. H. Benaissa, A. A. Alfaris, R. S. Aljumaah, S. M. Elnakhla, M. M. I. Salem, I. A. Ishag, M. El Khasmi and A. Beja-Pereira	https://doi.org/10.1016/j.livsci.2017.09.017
**2017**	Milk protein polymorphisms and casein haplotypes in Butana cattle	J Appl Genet	A. S. Ahmed, S. Rahmatalla, R. Bortfeldt, D. Arends, M. Reissmann and G. A. Brockmann	10.1007/s13353-016-0381-2
**2017**	Mitogenome Sequencing in the Genus Camelus Reveals Evidence for Purifying Selection and Long-term Divergence between Wild and Domestic Bactrian Camels	Sci Rep	E. Mohandesan, R. R. Fitak, J. Corander, A. Yadamsuren, B. Chuluunbat, O. Abdelhadi, A. Raziq, P. Nagy, G. Stalder, C. Walzer, B. Faye and P. A. Burger	10.1038/s41598-017-08995-8
**2017**	Molecular surveillance of Theileria parasites of livestock in Oman	Ticks Tick Borne Dis	A. Al-Fahdi, B. Alqamashoui, S. Al-Hamidhi, O. Kose, M. H. Tageldin, P. Bobade, E. H. Johnson, A. R. Hussain, T. Karagenc, A. Tait, B. Shiels, H. B. Bilgic and H. Babiker	10.1016/j.ttbdis.2017.05.008
**2017**	Monoallelic characteristic-bearing heterozygous L1053X in BRCA2 gene among Sudanese women with breast cancer	BMC Med Genet	A. A. Elimam, M. Aabdein, M. E. M. Eldeen, H. N. Altayb, M. A. Taha, M. N. Nimir, M. D. Dafaalla, M. M. Alfaki, M. A. Abdelrahim, A. A. Abdalla, M. I. Mohammed, M. Ellaithi, M. M. A. Hamid and M. A. S. Hassan	10.1186/s12881-017-0448-x
**2017**	New species in the papaya ringspot virus cluster: Insights into the evolution of the PRSV lineage	Virus Res	C. Desbiez, C. Wipf-Scheibel, P. Millot, E. Verdin, G. Dafalla and H. Lecoq	10.1016/j.virusres.2017.06.022
**2017**	Phenotypic and Genotypic Analysis of Multidrug-Resistant Mycobacterium tuberculosis Isolates from Sudanese Patients	Tuberc Res Treat	S. M. Sabeel, M. A. Salih, M. Ali, S. E. El-Zaki, N. Abuzeid, Z. A. Elgadi, H. N. Altayb, A. M. Elegail, N. Y. Ibrahim and B. K. Elamin	10.1155/2017/8340746
**2017**	Prevalence of current patterns and predictive trends of multidrug-resistant Salmonella Typhi in Sudan	Ann Clin Microbiol Antimicrob	A. A. Elshayeb, A. A. Ahmed, M. A. El Siddig and A. A. El Hussien	10.1186/s12941-017-0247-4
**2017**	Putative periodontal pathogens in the subgingival plaque of Sudanese subjects with aggressive periodontitis	Archives of Oral Biology	N. T. Hashim, G. J. Linden, L. Winning, M. E. Ibrahim, B. G. Gismalla, F. T. Lundy and I. A. El Karim	https://doi.org/10.1016/j.archoralbio.2017.04.027
**2017**	Pyrrolizines: Design, synthesis, anticancer evaluation and investigation of the potential mechanism of action	Bioorg Med Chem	A. M. Gouda, A. H. Abdelazeem, H. A. Omar, A. N. Abdalla, M. A. S. Abourehab and H. I. Ali	10.1016/j.bmc.2017.08.039
**2017**	Quadriacanthus species (Monogenea: Dactylogyridae) from catfishes (Teleostei: Siluriformes) in eastern Africa: new species, new records and first insights into interspecific genetic relationships	Parasit Vectors	K. Francová, M. Seifertová, R. Blažek, M. Gelnar, Z. N. Mahmoud and E. Řehulková	10.1186/s13071-017-2223-4

**2017**	Quantum probability ranking principle for ligand-based virtual screening	J Comput Aided Mol Des	M. M. Al-Dabbagh, N. Salim, M. Himmat, A. Ahmed and F. Saeed	10.1007/s10822-016-0003-4
**2017**	RNAi-mediated resistance to rice black-streaked dwarf virus in transgenic rice	Transgenic Res	M. M. Ahmed, S. Bian, M. Wang, J. Zhao, B. Zhang, Q. Liu, C. Zhang, S. Tang, M. Gu and H. Yu	10.1007/s11248-016-9999-4
**2017**	Role of outer membrane protein T in pathogenicity of avian pathogenic Escherichia coli	Res Vet Sci	H. M. A. Hejair, J. Ma, Y. Zhu, M. Sun, W. Dong, Y. Zhang, Z. Pan, W. Zhang and H. Yao	10.1016/j.rvsc.2017.01.026
**2017**	Spectrophotometric and molecular modelling studies on in vitro interaction of tyrosine kinase inhibitor linifanib with bovine serum albumin	PLoS One	T. A. Wani, A. H. Bakheit, S. Zargar, M. A. Hamidaddin and I. A. Darwish	10.1371/journal.pone.0176015
**2017**	The evaluation of GM6-based ELISA and ICT as diagnostic methods on a Mongolian farm with an outbreak of non-tsetse transmitted horse trypanosomosis	Vet Parasitol	B. Davaasuren, T. Amgalanbaatar, S. P. Musinguzi, K. Suganuma, D. Otgonsuren, E. Mossaad, S. Narantsatsral, B. Battur, B. Battsetseg, X. Xuan and N. Inoue	10.1016/j.vetpar.2017.07.036
**2017**	The genome landscape of indigenous African cattle	Genome Biol	J. Kim, O. Hanotte, O. A. Mwai, T. Dessie, S. Bashir, B. Diallo, M. Agaba, K. Kim, W. Kwak, S. Sung, M. Seo, H. Jeong, T. Kwon, M. Taye, K. D. Song, D. Lim, S. Cho, H. J. Lee, D. Yoon, S. J. Oh, S. Kemp, H. K. Lee and H. Kim	10.1186/s13059-017-1153-y
**2017**	The historical spread of Arabian Pastoralists to the eastern African Sahel evidenced by the lactase persistence -13,915*G allele and mitochondrial DNA	Am J Hum Biol	E. Priehodová, F. Austerlitz, M. Čížková, M. G. Mokhtar, E. S. Poloni and V. Černý	10.1002/ajhb.22950
**2017**	Three new cyclotetrapeptides isolated from Streptomyces sp. 447	Nat Prod Res	M. A. Abdalla	10.1080/14786419.2016.1263849
**2017**	Transcriptional changes of rice in response to rice black-streaked dwarf virus	Gene	M. M. S. Ahmed, W. Ji, M. Wang, S. Bian, M. Xu, W. Wang, J. Zhang, Z. Xu, M. Yu, Q. Liu, C. Zhang, H. Zhang, S. Tang, M. Gu and H. Yu	10.1016/j.gene.2017.07.015
**2017**	Trypanosoma vivax is the second leading cause of camel trypanosomosis in Sudan after Trypanosoma evansi	Parasit Vectors	E. Mossaad, B. Salim, K. Suganuma, P. Musinguzi, M. A. Hassan, E. A. Elamin, G. E. Mohammed, A. O. Bakhiet, X. Xuan, R. A. Satti and N. Inoue	10.1186/s13071-017-2117-5
**2017**	Whole genome population genetics analysis of Sudanese goats identifies regions harboring genes associated with major traits	BMC Genet	S. A. Rahmatalla, D. Arends, M. Reissmann, A. Said Ahmed, K. Wimmers, H. Reyer and G. A. Brockmann	10.1186/s12863-017-0553-z
**2018**	A Novel Missense Mutation in the SLC5A5 Gene in a Sudanese Family with Congenital Hypothyroidism	Thyroid	Y. Watanabe, R. S. Ebrhim, M. A. Abdullah and R. E. Weiss	10.1089/thy.2018.0137
**2018**	A population of wheat multiple synthetic derivatives: an effective platform to explore, harness and utilize genetic diversity of Aegilops tauschii for wheat improvement	Theor Appl Genet	Y. S. A. Gorafi, J. S. Kim, A. A. E. Elbashir and H. Tsujimoto	10.1007/s00122-018-3102-x
**2018**	Association of Copy Number Variation at Intron 3 of HMGA2 With Navel Length in Bos indicus	Front Genet	T. S. Aguiar, R. B. P. Torrecilha, M. Milanesi, A. T. H. Utsunomiya, B. B. Trigo, A. Tijjani, H. H.	10.3389/fgene.2018.00627

			Musa, F. L. Lopes, P. Ajmone-Marsan, R. Carvalheiro, H. H. R. Neves, A. S. do Carmo, O. Hanotte, T. S. Sonstegard, J. F. Garcia and Y. T. Utsunomiya	
**2018**	Association of toll-like receptor 2 polymorphisms with susceptibility to pulmonary tuberculosis in Sudanese	Egyptian Journal of Medical Human Genetics	H. Zaki, N. Gasmelseed, B. Abdalla and S. P. Yip	https://doi.org/10.1016/j.ejmhg.2018.01.001
**2018**	Biochemical and computational evaluation of Triptolide-induced cytotoxicity against NSCLC	Biomed Pharmacother	A. M. Hamdi, Z. Z. Jiang, M. Guerram, B. A. Yousef, H. M. Hassan, J. W. Ling and L. Y. Zhang	10.1016/j.biopha.2018.04.198
**2018**	Case report of a novel homozygous splice site mutation in PLA2G6 gene causing infantile neuroaxonal dystrophy in a Sudanese family	BMC Med Genet	L. E. O. Elsayed, I. N. Mohammed, A. A. A. Hamed, M. A. Elseed, M. A. M. Salih, A. Yahia, R. A. Siddig, M. Amin, M. Koko, M. I. Elbashir, M. E. Ibrahim, A. Brice, A. E. Ahmed and G. Stevanin	10.1186/s12881-018-0592-y
**2018**	Challenges imposed by minor reference alleles on the identification and reporting of clinical variants from exome data	BMC Genomics	M. Koko, M. O. E. Abdallah, M. Amin and M. Ibrahim	10.1186/s12864-018-4433-3
**2018**	Characterization of Pathogenic Bacteria Isolated from Sudanese Banknotes and Determination of Their Resistance Profile	Int J Microbiol	N. A. Abd Alfadil, M. Suliman Mohamed, M. M. Ali and E. A. I. El Nima	10.1155/2018/4375164
**2018**	Common microRNA⁻mRNA Interactions in Different Newcastle Disease Virus-Infected Chicken Embryonic Visceral Tissues	Int J Mol Sci	Y. Q. Jia, X. L. Wang, X. W. Wang, C. Q. Yan, C. J. Lv, X. Q. Li, Z. L. Chu, F. E. A. Adam, S. Xiao, S. X. Zhang and Z. Q. Yang	10.3390/ijms19051291
**2018**	Comparison of structures among Saccharomyces cerevisiae Grxs proteins	Genes Environ	M. Abdalla, W. A. Eltayb and A. Yousif	10.1186/s41021-018-0104-5
**2018**	Complete genome sequence of datura leaf curl virus, a novel begomovirus infecting Datura innoxia in Sudan, related to begomoviruses causing tomato yellow leaf curl disease	Arch Virol	H. S. Mohammed, M. A. El Siddig, A. A. El Hussein, J. Navas-Castillo and E. Fiallo-Olivé	10.1007/s00705-017-3574-z
**2018**	Cross sectional study to determine chloroquine resistance among Plasmodium falciparum clinical isolates from Khartoum, Sudan	F1000Res	W. S. Abdulla Mohammed, K. Yasin, N. S. Mahgoub and M. M. Abdel Hamid	10.12688/f1000research.13273.1
**2018**	DArTseq-based analysis of genomic relationships among species of tribe Triticeae	Sci Rep	O. U. Edet, Y. S. A. Gorafi, S. Nasuda and H. Tsujimoto	10.1038/s41598-018-34811-y
**2018**	Detection and molecular characterization of tick-borne pathogens infecting sheep and goats in Blue Nile and West Kordofan states in Sudan	Ticks and Tick-borne Diseases	S.-H. Lee, E. Mossaad, A. M. Ibrahim, A. A. Ismail, P. F. Adjou Moumouni, M. Liu, A. E. Ringo, Y. Gao, H. Guo, J. Li, A. Efstratiou, P. Musinguzi, T. E. E. Angara, K. Suganuma, N. Inoue and X. Xuan	https://doi.org/10.1016/j.ttbdis.2018.01.014
**2018**	Detection of Chlamydiaceae and Chlamydia-like organisms on the ocular surface of children and adults from a trachoma-endemic region	Sci Rep	E. Ghasemian, A. Inic-Kanada, A. Collingro, F. Tagini, E. Stein, H. Alchalabi, N. Schuerer, D. Keše, B. E. Babiker, N. Borel, G. Greub and T. Barisani-Asenbauer	10.1038/s41598-018-23887-1

**2018**	Developing reproducible bioinformatics analysis workflows for heterogeneous computing environments to support African genomics	BMC Bioinformatics	S. Baichoo, Y. Souilmi, S. Panji, G. Botha, A. Meintjes, S. Hazelhurst, H. Bendou, E. Beste, P. T. Mpangase, O. Souiai, M. Alghali, L. Yi, B. D. O'Connor, M. Crusoe, D. Armstrong, S. Aron, F. Joubert, A. E. Ahmed, M. Mbiyavanga, P. V. Heusden, L. E. Magosi, J. Zermeno, L. S. Mainzer, F. M. Fadlelmola, C. V. Jongeneel and N. Mulder	10.1186/s12859-018-2446-1
**2018**	Diversity pattern of Duffy binding protein sequence among Duffy-negatives and Duffy-positives in Sudan	Malar J	M. R. Hoque, M. M. A. Elfaki, M. A. Ahmed, S. K. Lee, F. Muh, M. M. Ali Albsheer, M. M. A. Hamid and E. T. Han	10.1186/s12936-018-2425-z
**2018**	EBV Associated Breast Cancer Whole Methylome Analysis Reveals Viral and Developmental Enriched Pathways	Front Oncol	M. O. E. Abdallah, U. K. Algizouli, M. A. Suliman, R. A. Abdulrahman, M. Koko, G. Fessahaye, J. H. Shakir, A. H. Fahal, A. M. Elhassan, M. E. Ibrahim and H. S. Mohamed	10.3389/fonc.2018.00316
**2018**	Efficient anchoring of alien chromosome segments introgressed into bread wheat by new Leymus racemosus genome-based markers	BMC Genet	O. U. Edet, J. S. Kim, M. Okamoto, K. Hanada, T. Takeda, M. Kishii, Y. S. A. Gorafi and H. Tsujimoto	10.1186/s12863-018-0603-1
**2018**	Epitope-Based Peptide Vaccine Against Fructose-Bisphosphate Aldolase of Madurella mycetomatis Using Immunoinformatics Approaches	Bioinform Biol Insights	A. A. Mohammed, A. L. AM, S. M. Sabeel, F. M. AbdElmarouf, A. I. Dirar, M. M. Ali, M. A. Khandgawi, A. M. Yousif, E. M. Abdulgadir, M. A. Sabahalkhair, A. E. Abbas and M. A. Hassan	10.1177/1177932218809703
**2018**	Expanding the phenome and variome of skeletal dysplasia	Genet Med	S. Maddirevula, S. Alsahli, L. Alhabeeb, N. Patel, F. Alzahrani, H. E. Shamseldin, S. Anazi, N. Ewida, H. S. Alsaif, J. Y. Mohamed, A. M. Alazami, N. Ibrahim, F. Abdulwahab, M. Hashem, M. Abouelhoda, D. Monies, N. Al Tassan, M. Alshammari, A. Alsagheir, M. Z. Seidahmed, S. Sogati, M. S. Aglan, M. H. Hamad, M. A. Salih, A. A. Hamed, N. Alhashmi, A. Nabil, F. Alfadli, G. M. H. Abdel-Salam, H. Alkuraya, W. O. Peitee, W. T. Keng, A. Qasem, A. M. Mushiba, M. S. Zaki, M. R. Fassad, M. Alfadhel, S. Alexander, Y. Sabr, S. Temtamy, A. V. Ekbote, S. Ismail, G. A. Hosny, G. A. Otaify, K. Amr, S. Al Tala, A. O. Khan, T. Rizk, A. Alaqeel, A. Alsiddiky, A. Singh, S. Kapoor, A. Alhashem, E. Faqeih, R. Shaheen and F. S. Alkuraya	10.1038/gim.2018.50

**2018**	First detection of Echinococcus granulosus sensu stricto (G1) in dogs in central Sudan	Parasitol Res	R. A. Omer, A. Daugschies, S. Gawlowska, A. Elnahas, P. Kern, S. Bashir, M. S. A. Ali, A. Osman and T. Romig	10.1007/s00436-018-5851-5
**2018**	First molecular characterization of Echinococcus granulosus (sensu stricto) genotype 1 among cattle in Sudan	BMC Vet Res	M. E. Ahmed, B. Salim, M. P. Grobusch and I. E. Aradaib	10.1186/s12917-018-1348-9
**2018**	Frequency of mitochondrial m.1555A > G mutation in Syrian patients with non-syndromic hearing impairment	BMC Ear Nose Throat Disord	H. Kaheel, A. Breß, M. A. Hassan, A. A. Shah, M. Amin, Y. H. Y. Bakhit and M. Kniper	10.1186/s12901-018-0055-2
**2018**	Fusarium metavorans sp. nov.: The frequent opportunist 'FSSC6'	Med Mycol	A. M. S. Al-Hatmi, S. A. Ahmed, A. D. van Diepeningen, M. Drogari-Apiranthitou, P. E. Verweij, J. F. Meis and G. S. de Hoog	10.1093/mmy/myx107
**2018**	Genetic characterization of extraintestinal Escherichia coli isolates from chicken, cow and swine	AMB Express	L. Chen, L. Wang, A. K. Yassin, J. Zhang, J. Gong, K. Qi, R. R. Ganta, Y. Zhang, Y. Yang, X. Han and C. Wang	10.1186/s13568-018-0646-8
**2018**	Genetic homogeneity of goat malaria parasites in Asia and Africa suggests their expansion with domestic goat host	Sci Rep	M. Kaewthamasorn, M. Takeda, T. Saiwichai, J. N. Gitaka, S. Tiawsirisup, Y. Imasato, E. Mossaad, A. Sarani, W. Kaewlamun, M. Channumsin, S. Chaiworakul, W. Katepongpun, S. Teeveerapunya, J. Panthong, D. K. Mureithi, S. Bawm, L. L. Htun, M. M. Win, A. A. Ismail, A. M. Ibrahim, K. Suganuma, H. Hakimi, R. Nakao, K. Katakura, M. Asada and O. Kaneko	10.1038/s41598-018-24048-0
**2018**	Genetic polymorphism in Hsp90AA1 gene is associated with the thermotolerance in Chinese Holstein cows	Cell Stress Chaperones	T. M. Badri, K. L. Chen, M. A. Alsiddig, L. Li, Y. Cai and G. L. Wang	10.1007/s12192-017-0873-y
**2018**	Genome-wide association study of body morphological traits in Sudanese goats	Anim Genet	S. A. Rahmatalla, D. Arends, M. Reissmann, K. Wimmers, H. Reyer and G. A. Brockmann	10.1111/age.12686
**2018**	Genome-wide characterization, identification, and expression analysis of the WD40 protein family in cotton	Genome	H. Salih, W. Gong, M. Mkulama and X. Du	10.1139/gen-2017-0237
**2018**	Genome-Wide Variation, Candidate Regions and Genes Associated With Fat Deposition and Tail Morphology in Ethiopian Indigenous Sheep	Front Genet	A. Ahbara, H. Bahbahani, F. Almathen, M. Al Abri, M. O. Agoub, A. Abeba, A. Kebede, H. H. Musa, S. Mastrangelo, F. Pilla, E. Ciani, O. Hanotte and J. M. Mwacharo	10.3389/fgene.2018.00699
**2018**	Hackathons as a means of accelerating scientific discoveries and knowledge transfer	Genome Res	A. Ghouila, G. H. Siwo, J. D. Entfellner, S. Panji, K. A. Button-Simons, S. Z. Davis, F. M. Fadlelmola, M. T. Ferdig and N. Mulder	10.1101/gr.228460.117
**2018**	Impact of PYROXD1 deficiency on cellular respiration and correlations with genetic analyses of limb-girdle muscular dystrophy in Saudi Arabia and Sudan	Physiol Genomics	M. Saha, H. M. Reddy, M. A. Salih, E. Estrella, M. D. Jones, S. Mitsuhashi, K. A. Cho, S. Suzuki-Hatano, S. A. Rizzo, M. H. Hamad, M. M. Mukhtar, A. A. Hamed, M. A. Elseed, M. Lek, E.	10.1152/physiolgenomics.00036.2018

			Valkanas, D. G. MacArthur, L. M. Kunkel, C. A. Pacak, I. Draper and P. B. Kang	
**2018**	In silico toxicity profiling of natural product compound libraries from African flora with anti-malarial and anti-HIV properties	Computational Biology and Chemistry	P. A. Onguéné, C. V. Simoben, G. W. Fotso, K. Andrae-Marobela, S. A. Khalid, B. T. Ngadjui, L. M. a. Mbaze and F. Ntie-Kang	https://doi.org/10.1016/j.compbiolchem.2017.12.002
**2018**	Indole Derivatives as Cyclooxygenase Inhibitors: Synthesis, Biological Evaluation and Docking Studies	Molecules	M. A. Bhat, M. A. Al-Omar, M. Raish, M. A. Ansari, H. A. Abuelizz, A. H. Bakheit and A. M. Naglah	10.3390/molecules23061250
**2018**	Intra-familial phenotypic heterogeneity in a Sudanese family with DARS2-related leukoencephalopathy, brainstem and spinal cord involvement and lactate elevation: a case report	BMC Neurol	A. Yahia, L. Elsayed, A. Babai, M. A. Salih, S. M. El-Sadig, M. Amin, M. Koko, R. Abubakr, R. Idris, S. Taha, S. A. Elmalik, A. Brice, A. E. Ahmed and G. Stevanin	10.1186/s12883-018-1180-7
**2018**	Leishmania Genome Dynamics during Environmental Adaptation Reveal Strain-Specific Differences in Gene Copy Number Variation, Karyotype Instability, and Telomeric Amplification	mBio	G. Bussotti, E. Gouzelou, M. Côrtes Boité, I. Kherachi, Z. Harrat, N. Eddaikra, J. C. Mottram, M. Antoniou, V. Christodoulou, A. Bali, F. Z. Guerfali, D. Laouini, M. Mukhtar, F. Dumetz, J. C. Dujardin, D. Smirlis, P. Lechat, P. Pescher, A. El Hamouchi, M. Lemrani, C. Chicharro, I. P. Llanes-Acevedo, L. Botana, I. Cruz, J. Moreno, F. Jeddi, K. Aoun, A. Bouratbine, E. Cupolillo and G. F. Späth	10.1128/mBio.01399-18
**2018**	Metabolomic Analysis of Pollen Grains with Different Germination Abilities from Two Clones of Chinese Fir (Cunninghamia lanceolata (Lamb) Hook)	Molecules	S. Fragallah, P. Wang, N. Li, Y. Chen and S. Lin	10.3390/molecules23123162
**2018**	miR-199a-3p Modulates MTOR and PAK4 Pathways and Inhibits Tumor Growth in a Hepatocellular Carcinoma Transgenic Mouse Model	Molecular Therapy - Nucleic Acids	E. Callegari, L. D'Abundo, P. Guerriero, C. Simioni, B. K. Elamin, M. Russo, A. Cani, C. Bassi, B. Zagatti, L. Giacomelli, S. Blandamura, F. Moshiri, S. Ultimo, A. Frassoldati, G. Altavilla, L. Gramantieri, L. M. Neri, S. Sabbioni and M. Negrini	https://doi.org/10.1016/j.omtn.2018.04.002
**2018**	Molecular and genetic characterization of peste des petits ruminants virus Kurdistan 2011 strain based on the haemagglutinin and fusion protein genes sequences	Small Ruminant Research	N. A. Osman, J. Veits and G. M. Keil	https://doi.org/10.1016/j.smallrumres.2018.08.007
**2018**	Molecular and in-silico analysis of single nucleotide polymorphism targeting human TP53 gene exon 5-8 in Sudanese esophageal cancer patients	F1000Res	R. M. Elfaki, M. S. Abdelaziz, H. N. Altayb, M. M. Munsoor and A. A. Gameel	10.12688/f1000research.15534.1
**2018**	Molecular detection and characterization of Theileria spp. infecting cattle in Sennar State, Sudan	Parasitol Res	S. B. Mohamed, A. Alagib, T. B. AbdElkareim, M. M. Hassan, W. C. Johnson, H. E. Hussein, N. S. Taus and M. W. Ueti	10.1007/s00436-018-5775-0

**2018**	Molecular phylogeny based on six nuclear genes suggests that Echinococcus granulosus sensu lato genotypes G6/G7 and G8/G10 can be regarded as two distinct species	Parasitology	T. Laurimäe, L. Kinkar, E. Moks, T. Romig, R. A. Omer, A. Casulli, G. Umhang, G. Bagrade, M. Irshadullah, M. Sharbatkhori, H. Mirhendi, F. Ponce-Gordo, S. V. Soriano, A. Varcasia, M. Rostami-Nejad, V. Andresiuk and U. Saarma	10.1017/s0031182018000719
**2018**	Molecular Survey of Viral and Bacterial Causes of Childhood Diarrhea in Khartoum State, Sudan	Front Microbiol	M. A. Adam, J. Wang, K. A. Enan, H. Shen, H. Wang, A. R. El Hussein, A. B. Musa, I. M. Khidir and X. Ma	10.3389/fmicb.2018.00112
**2018**	Nigrograna mackinnonii, Not Trematosphaeria grisea (syn., Madurella grisea), Is the Main Agent of Black Grain Eumycetoma in Latin America	J Clin Microbiol	S. A. Ahmed, G. M. González, A. Tirado-Sánchez, L. M. Moreno-López, S. de Hoog and A. Bonifaz	10.1128/jcm.01723-17
**2018**	Novel molecular marker-assisted strategy for production of wheat-Leymus mollis chromosome addition lines	Sci Rep	O. U. Edet, Y. S. A. Gorafi, S. W. Cho, M. Kishii and H. Tsujimoto	10.1038/s41598-018-34545-x
**2018**	Organizing and running bioinformatics hackathons within Africa: The H3ABioNet cloud computing experience	AAS Open Res	A. E. Ahmed, P. T. Mpangase, S. Panji, S. Baichoo, Y. Souilmi, F. M. Fadlelmola, M. Alghali, S. Aron, H. Bendou, E. De Beste, M. Mbiyavanga, O. Souiai, L. Yi, J. Zermeno, D. Armstrong, B. D. O'Connor, L. S. Mainzer, M. R. Crusoe, A. Meintjes, P. Van Heusden, G. Botha, F. Joubert, C. V. Jongeneel, S. Hazelhurst and N. Mulder	10.12688/aasopenres.12847.1
**2018**	PAIPline: pathogen identification in metagenomic and clinical next generation sequencing samples	Bioinformatics	A. Andrusch, P. W. Dabrowski, J. Klenner, S. H. Tausch, C. Kohl, A. A. Osman, B. Y. Renard and A. Nitsche	10.1093/bioinformatics/bty595
**2018**	Papillomavirus Infection in Humans and Dromedary Camels in Eastern Sudan	Vector Borne Zoonotic Dis	A. I. Khalafalla, A. Rector and A. K. Elfadl	10.1089/vbz.2017.2242
**2018**	Pharmacogenomics in diabetes: outcomes of thiamine therapy in TRMA syndrome	Diabetologia	A. M. Habeb, S. E. Flanagan, M. A. Zulali, M. A. Abdullah, R. Pomahačová, V. Boyadzhiev, L. E. Colindres, G. V. Godoy, T. Vasanthi, R. Al Saif, A. Setoodeh, A. Haghighi, A. Haghighi, Y. Shaalan, A. T. Hattersley, S. Ellard and E. De Franco	10.1007/s00125-018-4554-x
**2018**	Potential Deoxycytidine Kinase Inhibitory Activity of Amaryllidaceae Alkaloids: An In Silico Approach	J Pharm Bioallied Sci	B. A. Yousef, A. I. Dirar, M. A. A. Elbadawi, M. K. Awadalla and M. A. Mohamed	10.4103/jpbs.JPBS_44_18
**2018**	Probiotic Properties of Exopolysaccharide-Producing Lactobacillus Strains Isolated from Tempoyak	Molecules	E. S. Khalil, M. Y. Abd Manap, S. Mustafa, A. M. Alhelli and P. Shokryazdan	10.3390/molecules23020398
**2018**	Signatures of positive selection in African Butana and Kenana dairy zebu cattle	PLoS One	H. Bahbahani, B. Salim, F. Almathen, F. Al Enezi, J. M. Mwacharo and O. Hanotte	10.1371/journal.pone.0190446

**2018**	Structure analysis of yeast glutaredoxin Grx6 protein produced in Escherichia coli	Genes Environ	M. Abdalla, W. A. Eltayb, A. A. El-Arabey, R. Mo, T. I. M. Dafaalla, H. I. Hamouda, E. A. Bhat, A. Awadasseid and H. A. A. Ali	10.1186/s41021-018-0103-6
**2018**	Synthesis, Anti-Inflammatory Activity, and In Silico Study of Novel Diclofenac and Isatin Conjugates	Int J Med Chem	M. M. Ibrahim, T. Elsaman and M. Y. Al-Nour	10.1155/2018/9139786
**2018**	The 'forma specialis' issue in Fusarium: A case study in Fusarium solani f. sp. pisi	Sci Rep	A. Šišić, J. Baćanović-Šišić, A. M. S. Al-Hatmi, P. Karlovsky, S. A. Ahmed, W. Maier, G. S. de Hoog and M. R. Finckh	10.1038/s41598-018-19779-z
**2018**	The bacteriome at the onset of type 1 diabetes: A study from four geographically distant African and Asian countries	Diabetes Res Clin Pract	O. Cinek, L. Kramna, K. Mazankova, R. Odeh, A. Alassaf, M. U. Ibekwe, G. Ahmadov, B. M. E. Elmahi, H. Mekki, J. Lebl and M. A. Abdullah	10.1016/j.diabres.2018.08.010
**2018**	The benefits of analysing complete mitochondrial genomes: Deep insights into the phylogeny and population structure of Echinococcus granulosus sensu lato genotypes G6 and G7	Infect Genet Evol	T. Laurimäe, L. Kinkar, T. Romig, R. A. Omer, A. Casulli, G. Umhang, R. B. Gasser, A. Jabbar, M. Sharbatkhori, H. Mirhendi, F. Ponce-Gordo, L. E. Lazzarini, S. V. Soriano, A. Varcasia, M. Rostami Nejad, V. Andresiuk, P. Maravilla, L. M. González, M. Dybicz, J. Gawor, M. Šarkūnas, V. Šnábel, T. Kuzmina and U. Saarma	10.1016/j.meegid.2018.06.016
**2018**	The damage risk evaluation of Aphis gossypii on wheat by host shift and fitness comparison in wheat and cotton	Journal of Integrative Agriculture	Y.-j. Fan, F. Li, A. A. A. H. Mohammed, X.-q. Yi, M. Zhang, N. Desneux and X.-w. Gao	https://doi.org/10.1016/S2095-3119(17)61784-2
**2018**	The influence of microbial communities for triadimefon enantiomerization in soils with different pH values	Chirality	K. Mohamed Ahmed Talab, Z. H. Yang, J. H. Li, Y. Zhao, S. Alrasheed Mohamed Omer and Y. B. Xiong	10.1002/chir.22796
**2018**	The phylogeography and incidence of multi-drug resistant typhoid fever in sub-Saharan Africa	Nat Commun	S. E. Park, D. T. Pham, C. Boinett, V. K. Wong, G. D. Pak, U. Panzner, L. M. C. Espinoza, V. von Kalckreuth, J. Im, H. Schütt-Gerowitt, J. A. Crump, R. F. Breiman, Y. Adu-Sarkodie, E. Owusu-Dabo, R. Rakotozandrindrainy, A. B. Soura, A. Aseffa, N. Gasmelseed, K. H. Keddy, J. May, A. G. Sow, P. Aaby, H. M. Biggs, J. T. Hertz, J. M. Montgomery, L. Cosmas, B. Olack, B. Fields, N. Sarpong, T. J. L. Razafindrabe, T. M. Raminosoa, L. P. Kabore, E. Sampo, M. Teferi, B. Yeshitela, M. A. El Tayeb, A. Sooka, C. G. Meyer, R. Krumkamp, D. M. Dekker, A. Jaeger, S. Poppert, A. Tall, A. Niang, M. Bjerregaard-Andersen, S. V. Løfberg, H. J. Seo, H. J. Jeon, J. F. Deerin, J. Park, F. Konings, M. Ali, J. D. Clemens, P. Hughes, J. N. Sendagala, T. Vudriko, R. Downing, U. N. Ikumapayi, G. A.	10.1038/s41467-018-07370-z

			Mackenzie, S. Obaro, S. Argimon, D. M. Aanensen, A. Page, J. A. Keane, S. Duchene, Z. Dyson, K. E. Holt, G. Dougan, F. Marks and S. Baker	
**2018**	Theileria lestoquardi in Sudan is highly diverse and genetically distinct from that in Oman	Infect Genet Evol	H. Awad, S. Al-Hamidhi, A. M. El Hussein, Y. M. Z. Yousif, K. M. Taha, D. A. Salih, W. Weir and H. A. Babiker	10.1016/j.meegid.2018.04.014
**2018**	Two new species of the Fusarium solani species complex isolated from compost and hibiscus (Hibiscus sp.)	Antonie Van Leeuwenhoek	A. Šišić, A. M. S. Al-Hatmi, J. Baćanović-Šišić, S. A. Ahmed, D. Dennenmoser, G. S. de Hoog and M. R. Finckh	10.1007/s10482-018-1068-y
**2018**	Unraveling the binding characteristics of the anti-HIV agents abacavir, efavirenz and emtricitabine to bovine serum albumin using spectroscopic and molecular simulation approaches	Journal of Molecular Liquids	A. M. Alanazi, A. S. Abdelhameed, A. H. Bakheit, F. M. Almutairi, A. Alkhider, R. N. Herqash and I. A. Darwish	https://doi.org/10.1016/j.molliq.2017.12.066
**2019**	Absence of K13 gene mutations among artesunate/sulfadoxine-pyrimethamine treatment failures of Sudanese Plasmodium falciparum isolates from Damazin, southeast Sudan	Trans R Soc Trop Med Hyg	M. M. Abdel Hamid, W. M. E. Abdallah, M. Hussien, N. M. Mohammed, E. M. Malik, M. E. Ahmed and A. O. Mohamed	10.1093/trstmh/trz027
**2019**	Analysis of hepatitis B virus-mixed genotype infection by ultra deep pyrosequencing in Sudanese patients, 2015-2016	Infection	K. A. Enan, C. Minosse, A. R. M. El Hussein, M. Selleri, E. Giombini, M. R. Capobianchi, I. M. Elkhidir, M. O. Mustafa, O. M. Khair, D. A. Hassan and A. R. Garbuglia	10.1007/s15010-019-01306-5
**2019**	Analysis of nad2 and nad5 enables reliable identification of genotypes G6 and G7 within the species complex Echinococcus granulosus sensu lato	Infect Genet Evol	T. Laurimäe, L. Kinkar, T. Romig, G. Umhang, A. Casulli, R. A. Omer, M. Sharbatkhori, H. Mirhendi, F. Ponce-Gordo, L. E. Lazzarini, S. V. Soriano, A. Varcasia, M. Rostami-Nejad, V. Andresiuk, P. Maravilla, L. M. González, M. Dybicz, J. Gawor, M. Šarkūnas, V. Šnábel, T. Kuzmina, E. B. Kia and U. Saarma	10.1016/j.meegid.2019.103941
**2019**	Antioxidant activities and molecular docking of 2-thioxobenzo[g]quinazoline derivatives	Pharmacol Rep	R. Al-Salahi, H. A. A. Taie, A. H. Bakheit, M. Marzouk, A. A. Almehizia, R. Herqash and H. A. Abuelizz	10.1016/j.pharep.2019.04.003
**2019**	Association of ESR1 polymorphisms (rs3020314 and rs1514348) with breast cancer in Sudanese women. A pilot study	Gene Reports	A. T. Gebreslasie, A. Faggad, H. Y. Zaki and B. E. Abdalla	https://doi.org/10.1016/j.genrep.2019.100396
**2019**	Casein Gene Cluster in Camelids: Comparative Genome Analysis and New Findings on Haplotype Variability and Physical Mapping	Front Genet	A. Pauciullo, E. T. Shuiep, M. D. Ogah, G. Cosenza, L. Di Stasio and G. Erhardt	10.3389/fgene.2019.00748
**2019**	Chaetomium atrobrunneum causing human eumycetoma: The first report	PLoS Negl Trop Dis	N. A. Mhmoud, A. Santona, M. Fiamma, E. E. Siddig, M. Deligios, S. M. Bakhiet, S. Rubino and A. H. Fahal	10.1371/journal.pntd.0007276

**2019**	Comparative transcriptome analysis of TUCPs in Gossypium hirsutum Ligon-lintless-1 mutant and their proposed functions in cotton fiber development	Mol Genet Genomics	H. Salih, W. Gong, S. He, N. S. Mustafa and X. Du	10.1007/s00438-018-1482-x
**2019**	Congenital Hypothyroidism due to Oligogenic Mutations in Two Sudanese Families	Thyroid	Y. Watanabe, R. J. Bruellman, R. S. Ebrhim, M. A. Abdullah, A. M. Dumitrescu, S. Refetoff and R. E. Weiss	10.1089/thy.2018.0295
**2019**	Crimean-Congo haemorrhagic fever virus in Hyalomma impeltatum ticks from North Kordofan, the Sudan	Int J Infect Dis	L. Chitimia-Dobler, M. H. Issa, M. E. Ezalden, I. A. Yagoub, M. A. Abdalla, A. O. Bakhiet, S. Schaper, R. Rieß, P. Vollmar, A. Grumbach, M. Bestehorn, M. Antwerpen, G. Dobler and Y. A. Shuaib	10.1016/j.ijid.2019.09.012
**2019**	Cytotoxicity of cucurbitacin E from Citrullus colocynthis against multidrug-resistant cancer cells	Phytomedicine	M. E. M. Saeed, J. C. Boulos, G. Elhaboub, D. Rigano, A. Saab, M. R. Loizzo, L. E. A. Hassan, Y. Sugimoto, S. Piacente, R. Tundis, S. Yagi, H. Khalid and T. Efferth	10.1016/j.phymed.2019.152945
**2019**	Detection of Micro-invasion in Sudanese Oral Verrucous Carcinoma Samples Using Syndecan-1 Stain	Biomark Cancer	A. Elhassan, A. M. Suleiman, N. El Dawi and S. B. Mohamed	10.1177/1179299x19861957
**2019**	Distribution of Duffy Phenotypes among Plasmodium vivax Infections in Sudan	Genes (Basel)	M. M. A. Albsheer, K. Pestana, S. Ahmed, M. Elfaki, E. Gamil, S. M. Ahmed, M. E. Ibrahim, A. M. Musa, E. Lo and M. M. A. Hamid	10.3390/genes10060437
**2019**	Epidemiological and molecular investigation of resurgent cutaneous leishmaniasis in Sudan	Int J Infect Dis	S. Collis, S. El-Safi, A. A. Atia, T. Bhattacharyya, A. Hammad, M. Den Boer, H. Le, J. A. Whitworth and M. A. Miles	10.1016/j.ijid.2019.08.018
**2019**	Evidence of multiple point mutations in Theileria annulata cytochrome b gene incriminated in buparvaquone treatment failure	Acta Trop	E. Chatanga, E. Mosssad, H. Abdo Abubaker, S. Amin Alnour, K. Katakura, R. Nakao and B. Salim	10.1016/j.actatropica.2018.12.041
**2019**	First detection and genetic characterization of peste des petits ruminants virus from dorcas gazelles "Gazella dorcas" in the Sudan, 2016-2017	Arch Virol	R. M. Asil, M. Ludlow, A. Ballal, S. Alsarraj, W. H. Ali, B. A. Mohamed, S. M. Mutwakil and N. A. Osman	10.1007/s00705-019-04330-w
**2019**	First report and molecular characterization of Cryptosporidium spp. in humans and animals in Khartoum state, Sudan	Vet World	K. Y. Adam, A. A. Ismail, M. A. Masri and A. A. Gameel	10.14202/vetworld.2019.183-189
**2019**	First Whole-Genome Sequence of a Highly Resistant Klebsiella pneumoniae Sequence Type 14 Strain Isolated from Sudan	Microbiol Resour Announc	S. B. Mohamed, S. Kambal, A. Munir, N. Abdalla, M. Hassan, A. Hamad, S. Mohammed, F. Ahmed, O. Hamid, A. Ismail and M. Allam	10.1128/mra.00552-19
**2019**	Frequent expansion of Plasmodium vivax Duffy Binding Protein in Ethiopia and its epidemiological significance	PLoS Negl Trop Dis	E. Lo, J. B. Hostetler, D. Yewhalaw, R. D. Pearson, M. M. A. Hamid, K. Gunalan, D. Kepple, A. Ford, D. A. Janies, J. C. Rayner, L. H. Miller and G. Yan	10.1371/journal.pntd.0007222
**2019**	From taxonomic deflation to newly detected cryptic species: Hidden diversity in a widespread African squeaker catfish	Sci Rep	D. Jirsová, J. Štefka, R. Blažek, J. O. Malala, D. E. Lotuliakou, Z. N. Mahmoud and M. Jirků	10.1038/s41598-019-52306-2

**2019**	Genetic diversity analysis of fourteen geese breeds based on str genotyping technique	Asian-Australas J Anim Sci	H. Abdel Moniem, Y. Yao Zong, A. Abdallah and G. H. Chen	10.5713/ajas.18.0589
**2019**	Genetic polymorphism of the N-terminal region in circumsporozoite surface protein of Plasmodium falciparum field isolates from Sudan	Malar J	N. S. Mohamed, M. M. Ali Albsheer, H. Abdelbagi, E. E. Siddig, M. A. Mohamed, A. E. Ahmed, R. A. Omer, M. S. Muneer, A. Ahmed, H. A. Osman, M. S. Ali, I. M. Eisa and M. M. Elbasheir	10.1186/s12936-019-2970-0
**2019**	Genome Diversity and Signatures of Selection for Production and Performance Traits in Dromedary Camels	Front Genet	H. Bahbahani, H. H. Musa, D. Wragg, E. S. Shuiep, F. Almathen and O. Hanotte	10.3389/fgene.2019.00893
**2019**	Genome-wide analysis of cotton C2H2-zinc finger transcription factor family and their expression analysis during fiber development	BMC Plant Biol	H. Salih, M. R. Odongo, W. Gong, S. He and X. Du	10.1186/s12870-019-2003-8
**2019**	Genomic analysis of methicillin-resistant Staphylococcus aureus strain SO-1977 from Sudan	BMC Microbiol	M. S. Ali, N. M. Isa, F. M. Abedelrhman, T. B. Alyas, S. E. Mohammed, A. E. Ahmed, Z. S. A. Ahmed, N. S. Lau, M. I. Garbi, A. A. Amirul, A. O. Seed, R. A. Omer and S. B. Mohamed	10.1186/s12866-019-1470-2
**2019**	Genomic evidence for shared common ancestry of East African hunting-gathering populations and insights into local adaptation	Proc Natl Acad Sci U S A	L. B. Scheinfeldt, S. Soi, C. Lambert, W. Y. Ko, A. Coulibaly, A. Ranciaro, S. Thompson, J. Hirbo, W. Beggs, M. Ibrahim, T. Nyambo, S. Omar, D. Woldemeskel, G. Belay, A. Froment, J. Kim and S. A. Tishkoff	10.1073/pnas.1817678116
**2019**	Germline TP53 mutation spectrum in Sudanese premenopausal breast cancer patients: correlations with reproductive factors	Breast Cancer Res Treat	G. M. Aceto, K. D. Awadelkarim, M. Di Nicola, C. Moscatello, M. R. Pantalone, F. Verginelli, N. E. Elwali and R. Mariani-Costantini	10.1007/s10549-019-05168-1
**2019**	Global phylogeography and ancient evolution of the widespread human gut virus crAssphage	Nat Microbiol	R. A. Edwards, A. A. Vega, H. M. Norman, M. Ohaeri, K. Levi, E. A. Dinsdale, O. Cinek, R. K. Aziz, K. McNair, J. J. Barr, K. Bibby, S. J. J. Brouns, A. Cazares, P. A. de Jonge, C. Desnues, S. L. Díaz Muñoz, P. C. Fineran, A. Kurilshikov, R. Lavigne, K. Mazankova, D. T. McCarthy, F. L. Nobrega, A. Reyes Muñoz, G. Tapia, N. Trefault, A. V. Tyakht, P. Vinuesa, J. Wagemans, A. Zhernakova, F. M. Aarestrup, G. Ahmadov, A. Alassaf, J. Anton, A. Asangba, E. K. Billings, V. A. Cantu, J. M. Carlton, D. Cazares, G. S. Cho, T. Condeff, P. Cortés, M. Cranfield, D. A. Cuevas, R. De la Iglesia, P. Decewicz, M. P. Doane, N. J. Dominy, L. Dziewit, B. M. Elwasila, A. M. Eren, C. Franz, J. Fu, C. Garcia-Aljaro, E. Ghedin, K. M.	10.1038/s41564-019-0494-6

			Gulino, J. M. Haggerty, S. R. Head, R. S. Hendriksen, C. Hill, H. Hyöty, E. N. Ilina, M. T. Irwin, T. C. Jeffries, J. Jofre, R. E. Junge, S. T. Kelley, M. Khan Mirzaei, M. Kowalewski, D. Kumaresan, S. R. Leigh, D. Lipson, E. S. Lisitsyna, M. Llagostera, J. M. Maritz, L. C. Marr, A. McCann, S. Molshanski-Mor, S. Monteiro, B. Moreira-Grez, M. Morris, L. Mugisha, M. Muniesa, H. Neve, N. P. Nguyen, O. D. Nigro, A. S. Nilsson, T. O'Connell, R. Odeh, A. Oliver, M. Piuri, A. J. Prussin Ii, U. Qimron, Z. X. Quan, P. Rainetova, A. Ramírez-Rojas, R. Raya, K. Reasor, G. A. O. Rice, A. Rossi, R. Santos, J. Shimashita, E. N. Stachler, L. C. Stene, R. Strain, R. Stumpf, P. J. Torres, A. Twaddle, M. Ugochi Ibekwe, N. Villagra, S. Wandro, B. White, A. Whiteley, K. L. Whiteson, C. Wijmenga, M. M. Zambrano, H. Zschach and B. E. Dutilh	
**2019**	Human Leukocyte Antigen (HLA) class I and II datasets for sudanese	Data in Brief	A. M. Dafalla, H. A. Edinur, M. Abdelwahed, A. A. Elemam, A. A. Ibrahim, A. Mohamadani, M. Magzoub and G. ElGhazali	https://doi.org/10.1016/j.dib.2019.104027
**2019**	Immunoinformatics Approach for Multiepitope Vaccine Prediction from H, M, F, and N Proteins of Peste des Petits Ruminants Virus	J Immunol Res	B. B. M. Gaafar, S. A. Ali, K. A. Abd-Elrahman and Y. A. Almofti	10.1155/2019/6124030
**2019**	Immunoinformatics Approach for Multiepitopes Vaccine Prediction against Glycoprotein B of Avian Infectious Laryngotracheitis Virus	Adv Bioinformatics	S. A. Ali, Y. A. Almofti and K. A. Abd-Elrahman	10.1155/2019/1270485
**2019**	Improvement and use of CRISPR/Cas9 to engineer a sperm-marking strain for the invasive fruit pest Drosophila suzukii	BMC Biotechnol	H. M. M. Ahmed, L. Hildebrand and E. A. Wimmer	10.1186/s12896-019-0588-5
**2019**	In Silico Genetics Revealing 5 Mutations in CEBPA Gene Associated With Acute Myeloid Leukemia	Cancer Inform	M. I. Mustafa, Z. O. Mohammed, N. S. Murshed, N. M. Elfadol, A. H. Abdelmoneim and M. A. Hassan	10.1177/1176935119870817
**2019**	Interleukin-10 (1082G/A) Polymorphism is Associated with Susceptibility of Acute Myeloid Leukemia Patients in Sudanese Population	Asian Pac J Cancer Prev	O. M. Sharif, R. Hassan, A. A. Mohammed Basbaeen, A. H. Mohmed and I. K. Ibrahim	10.31557/apjcp.2019.20.7.1939
**2019**	Long non-coding RNAs and their potential functions in Ligon-lintless-1 mutant cotton during fiber development	BMC Genomics	H. Salih, W. Gong, S. He, W. Xia, M. R. Odongo and X. Du	10.1186/s12864-019-5978-5

**2019**	LXR/RXR pathway signaling associated with triple-negative breast cancer in African American women	Breast Cancer (Dove Med Press)	O. Torres-Luquis, K. Madden, M. N'Dri N, R. Berg, O. F. Olopade, W. Ngwa, D. Abuidris, S. Mittal, B. Lyn-Cook and S. I. Mohammed	10.2147/bctt.S185960
**2019**	Managing genomic variant calling workflows with Swift/T	PLoS One	A. E. Ahmed, J. Heldenbrand, Y. Asmann, F. M. Fadlelmola, D. S. Katz, K. Kendig, M. C. Kendzior, T. Li, Y. Ren, E. Rodriguez, M. R. Weber, J. M. Wozniak, J. Zermeno and L. S. Mainzer	10.1371/journal.pone.0211608
**2019**	Mutations in the TaPIN1 peptidyl prolyl isomerase gene in Theileria annulata parasites isolated in Sudan	International Journal for Parasitology: Drugs and Drug Resistance	B. Salim, E. Chatanga, G. Jannot, E. Mossaad, R. Nakao and J. B. Weitzman	https://doi.org/10.1016/j.ijpddr.2019.11.001
**2019**	Novel BTK inhibitor acalabrutinib (ACP-196) tightly binds to site I of the human serum albumin as observed by spectroscopic and computational studies	Int J Biol Macromol	A. S. Abdelhameed, A. M. Alanazi, A. H. Bakheit, E. S. Hassan, R. N. Herqash and F. M. Almutairi	10.1016/j.ijbiomac.2019.01.083
**2019**	Novel Deleterious nsSNPs within MEFV Gene that Could Be Used as Diagnostic Markers to Predict Hereditary Familial Mediterranean Fever: Using Bioinformatics Analysis	Adv Bioinformatics	M. I. Mustafa, T. A. Abdelhameed, F. A. Abdelrhman, S. A. Osman and M. A. Hassan	10.1155/2019/1651587
**2019**	Overlap of polymicrogyria, hydrocephalus, and Joubert syndrome in a family with novel truncating mutations in ADGRG1/GPR56 and KIAA0556	Neurogenetics	E. S. Cauley, A. Hamed, I. N. Mohamed, M. Elseed, S. Martinez, A. Yahia, F. Abozar, R. Abubakr, M. Koko, L. Elsayed, X. Piao, M. A. Salih and M. C. Manzini	10.1007/s10048-019-00577-2
**2019**	Potential role of dogs as sentinels and reservoirs for piroplasms infecting equine and cattle in Riyadh City, Saudi Arabia	Acta Trop	B. Salim, A. D. Alanazi, R. Omori, M. S. Alyousif, I. O. Alanazi, K. Katakura and R. Nakao	10.1016/j.actatropica.2019.02.029
**2019**	Proteomics and Docking Study Targeting Penicillin-Binding Protein and Penicillin-Binding Protein2a of Methicillin-Resistant Staphylococcus aureus Strain SO-1977 Isolated from Sudan	Evol Bioinform Online	S. B. Mohamed, T. A. Adlan, N. A. Khalafalla, N. I. Abdalla, Z. S. Ali, A. Munir Ka, M. M. Hassan and M. B. Elnour	10.1177/1176934319864945
**2019**	Rare variant in LAMA2 gene causing congenital muscular dystrophy in a Sudanese family. A case report	Acta Myol	M. Amin, Y. Bakhit, M. Koko, M. O. M. Ibrahim, M. A. Salih, M. Ibrahim and O. A. Seidi	PMID: 31309178
**2019**	Re-annotation for hypothetical protein CA803_03125 of Methicillin-Resistant Staphylococcus aureus strain SO-1977 isolated from Sudan	Bioinformation	S. B. Mohamed, M. M. Hassan, K. A. Munir, N. I. Abdalla, T. A. Adlan and A. K. Babiker	10.6026/97320630015160
**2019**	Recombination and purifying and balancing selection determine the evolution of major antigenic protein 1 (map 1) family genes in Ehrlichia ruminantium	Gene	B. Salim, M. Amin, M. Igarashi, K. Ito, F. Jongejan, K. Katakura, C. Sugimoto and R. Nakao	10.1016/j.gene.2018.10.028
**2019**	Refining wet lab experiments with in silico searches: A rational quest for diagnostic peptides in visceral leishmaniasis	PLoS Negl Trop Dis	B. C. Bremer Hinckel, T. Marlais, S. Airs, T. Bhattacharyya, H. Imamura, J. C. Dujardin, S. El-Safi, O. P. Singh, S. Sundar, A. K. Falconar, B. Andersson, S. Litvinov, M. A. Miles and P. Mertens	10.1371/journal.pntd.0007353

**2019**	Species of Characidotrema Paperna & Thurston, 1968 (Monogenea: Dactylogyridae) from fishes of the Alestidae (Characiformes) in Africa: new species, host-parasite associations and first insights into the phylogeny of the genus	Parasit Vectors	E. Řehulková, M. L. Kičinjaová, Z. N. Mahmoud, M. Gelnar and M. Seifertová	10.1186/s13071-019-3580-y
**2019**	Species-Specific Conservation of Linear Antigenic Sites on Vaccinia Virus A27 Protein Homologs of Orthopoxviruses	Viruses	H. P. Ahsendorf, L. L. Gan, K. H. Eltom, A. Abd El Wahed, S. K. Hotop, R. L. Roper, U. Beutling, M. Broenstrup, C. Stahl-Hennig, L. E. Hoelzle and C. P. Czerny	10.3390/v11060493
**2019**	Ten simple rules for organizing a webinar series	PLoS Comput Biol	F. M. Fadlelmola, S. Panji, A. E. Ahmed, A. Ghouila, W. A. Akurugu, J. B. Domelevo Entfellner, O. Souiai and N. Mulder	10.1371/journal.pcbi.1006671
**2019**	The prevalence of the culturable human skin aerobic bacteria in Riyadh, Saudi Arabia	BMC Microbiol	A. Shami, S. Al-Mijalli, P. Pongchaikul, A. Al-Barrag and S. AbduRahim	10.1186/s12866-019-1569-5
**2019**	VNTR confirms the heterogeneity of Madurella mycetomatis and is a promising typing tool for this mycetoma causing agent	Med Mycol	W. Lim, K. Eadie, D. Horst-Kreft, S. A. Ahmed, A. H. Fahal and W. W. J. van de Sande	10.1093/mmy/myy055
**2019**	Whole-Genome Sequence of Acinetobacter baumannii Strain NUBRI-A, Isolated from a Hospitalized Patient in Khartoum, Sudan	Microbiol Resour Announc	S. B. Mohamed, M. Hassan, A. Munir, S. Kambal, N. I. Abdalla, A. Hamad, S. Mohammed, F. Ahmed, O. Hamid, A. Ismail and M. Allam	10.1128/mra.00542-19
**2019**	Whole-Genome Sequence of High-Risk Clone Sequence Type 111 of Pseudomonas aeruginosa Strain NUBRI-P, Isolated from a Wounded Sudanese Patient	Microbiol Resour Announc	S. B. Mohamed, S. Kambal, A. Munir, N. I. Abdalla, A. Hamad, S. E. Mohammed, F. E. Ahmed, O. Hamid, M. M. Hassan, A. Ismail and M. Allam	10.1128/mra.00879-19
**2019**	Whole-genome sequencing of ocular Chlamydia trachomatis isolates from Gadarif State, Sudan	Parasit Vectors	A. A. I. Alkhidir, M. J. Holland, W. I. Elhag, C. A. Williams, J. Breuer, A. E. Elemam, K. M. K. El Hussain, M. E. H. Ournasseir and H. Pickering	10.1186/s13071-019-3770-7
**2020**	A Computational Vaccine Designing Approach for MERS-CoV Infections	Methods Mol Biol	H. S. Ibrahim and S. K. Kafi	10.1007/978-1-0716-0389-5_4
**2020**	Antigen gene and variable number tandem repeat (VNTR) diversity in Theileria parva parasites from Ankole cattle in south-western Uganda: Evidence for conservation in antigen gene sequences combined with extensive polymorphism at VNTR loci	Transbound Emerg Dis	A. Nanteza, I. Obara, P. Kasaija, E. Mwega, F. Kabi, D. A. Salih, M. Njahira, J. Njuguna, D. Odongo, R. P. Bishop, R. A. Skilton, J. Ahmed, P. H. Clausen and G. W. Lubega	10.1111/tbed.13311
**2020**	Assessment of Plasmodium falciparum drug resistance molecular markers from the Blue Nile State, Southeast Sudan	Malar J	A. O. Mohamed, M. Hussien, A. Mohamed, A. Suliman, N. S. Elkando, H. Abdelbagi, E. M. Malik, M. H. Abdelraheem and M. M. A. Hamid	10.1186/s12936-020-03165-0
**2020**	Association of VEGFA and IL1β gene polymorphisms with preeclampsia in Sudanese women	Mol Genet Genomic Med	H. M. Hamid, S. E. Abdalla, M. Sidig, I. Adam and H. Z. Hamdan	10.1002/mgg3.1119
**2020**	Bioassay-guided isolation of anti-hepatitis B virus flavonoid myricetin-3-O-rhamnoside along with quercetin from Guiera senegalensis leaves	Saudi Pharmaceutical Journal	M. K. Parvez, M. S. Al-Dosari, A. H. Arbab, A. J. Al-Rehaily and M. A. S. Abdelwahid	https://doi.org/10.1016/j.jsps.2020.03.006

**2020**	Central Congenital Hypothyroidism Caused by a Novel Mutation, C47W, in the Cysteine Knot Region of TSHβ	Horm Res Paediatr	R. S. Ebrhim, R. J. Bruellman, Y. Watanabe, M. K. Creech, M. A. Abdullah, A. M. Dumitrescu, S. Refetoff and R. E. Weiss	10.1159/000504981
**2020**	Chemical profile, antiproliferative, antioxidant, and enzyme inhibition activities and docking studies of Cymbopogon schoenanthus (L.) Spreng. and Cymbopogon nervatus (Hochst.) Chiov. from Sudan	J Food Biochem	S. Yagi, A. B. A. Mohammed, T. Tzanova, H. Schohn, H. Abdelgadir, A. Stefanucci, A. Mollica and G. Zengin	10.1111/jfbc.13107
**2020**	Computational determination of human PPARG gene: SNPs and prediction of their effect on protein functions of diabetic patients	Clin Transl Med	H. A. Mustafa, A. M. S. Albkrye, B. M. AbdAlla, M. A. M. Khair, N. Abdelwahid and H. A. Elnasri	10.1186/s40169-020-0258-1
**2020**	Delivering blended bioinformatics training in resource-limited settings: a case study on the University of Khartoum H3ABioNet node	Brief Bioinform	A. E. Ahmed, A. A. Awadallah, M. Tagelsir, M. A. Suliman, A. Eltigani, H. Elsafi, B. D. Hamdelnile, M. A. Mukhtar and F. M. Fadlelmola	10.1093/bib/bbz004
**2020**	Diagnostic Implications of Mycetoma Derived from Madurella pseudomycetomatis Isolates from Mexico	J Eur Acad Dermatol Venereol	B. Nyuykonge, C. H. W. Klaassen, W. H. A. Zandijk, G. S. de Hoog, S. A. Ahmed, M. Desnos-Ollivier, A. Verbon, A. Bonifaz and W. W. J. van de Sande	10.1111/jdv.16402
**2020**	Diversity and geographic distribution of soil streptomycetes with antagonistic potential against actinomycetoma-causing Streptomyces sudanensis in Sudan and South Sudan	BMC Microbiol	M. E. Hamid, T. Reitz, M. R. P. Joseph, K. Hommel, A. Mahgoub, M. M. Elhassan, F. Buscot and M. Tarkka	10.1186/s12866-020-1717-y
**2020**	Draft Genome Sequences of Three Clinical Isolates of Madurella mycetomatis, the Major Cause of Black-Grain Mycetoma	Microbiol Resour Announc	E. S. Khidir, A. Ahmed, A. H. Fahal and A. A. Ibrahim	10.1128/mra.01533-19
**2020**	Epitope-Based Peptide Vaccine against Glycoprotein G of Nipah Henipavirus Using Immunoinformatics Approaches	J Immunol Res	A. A. Mohammed, S. W. Shantier, M. I. Mustafa, H. K. Osman, H. E. Elmansi, I. A. Osman, R. A. Mohammed, F. A. Abdelrhman, M. E. Elnnewery, E. M. Yousif, M. M. Mustafa, N. M. Elfadol, A. I. Abdalla, E. Mahmoud, A. A. Yagaub, Y. A. Ahmed and M. A. Hassan	10.1155/2020/2567957
**2020**	Ethyl benzoate bearing pyrrolizine/indolizine moieties: Design, synthesis and biological evaluation of anti-inflammatory and cytotoxic activities	Bioorganic Chemistry	K. M. Attalah, A. N. Abdalla, A. Aslam, M. Ahmed, M. A. S. Abourehab, N. A. ElSawy and A. M. Gouda	https://doi.org/10.1016/j.bioorg.2019.103371
**2020**	Extensive In Silico Analysis of ATL1 Gene : Discovered Five Mutations That May Cause Hereditary Spastic Paraplegia Type 3A	Scientifica (Cairo)	M. I. Mustafa, N. S. Murshed, A. H. Abdelmoneim, M. I. Abdelmageed, N. M. Elfadol and A. M. Makhawi	10.1155/2020/8329286
**2020**	Field investigation and phylogenetic characterization of orf virus (ORFV) circulating in small ruminants and Pseudocowpoxvirus (PCPV) in dromedary camels of eastern Sudan	Heliyon	A. I. Khalafalla, A. E. Elhag and H. Z. A. Ishag	10.1016/j.heliyon.2020.e03595
**2020**	First report on molecular characterization and phylogenetic analysis of Reticuloendotheliosis virus in Sudan	Trop Anim Health Prod	S. H. Alfaki, M. O. Hussien, N. A. Osman, K. A. Enan and A. R. M. El Hussein	10.1007/s11250-020-02235-4

**2020**	Genetic Diversity and Sequence Polymorphism of Two Genes Encoding Theileria parva Antigens Recognized by CD8(+) T Cells among Vaccinated and Unvaccinated Cattle in Malawi	Pathogens	E. Chatanga, K. Hayashida, W. Muleya, K. Kusakisako, M. A. M. Moustafa, B. Salim, K. Katakura, C. Sugimoto, N. Nonaka and R. Nakao	10.3390/pathogens9050334
**2020**	Genome Sequence of Escherichia coli Clone O25:H4 Sequence Type 131, Isolated from a Sudanese Patient with Urinary Tract Infection	Microbiol Resour Announc	S. B. Mohamed, M. M. Hassan, S. Kambal, A. Munir, N. I. Abdalla, A. Hamad, S. E. Mohammed, F. E. Ahmed, O. Hamid, A. Ismail and M. Allam	10.1128/mra.01326-19
**2020**	Genomic Diversity, Population Structure, and Signature of Selection in Five Chinese Native Sheep Breeds Adapted to Extreme Environments	Genes (Basel)	A. Abied, A. Bagadi, F. Bordbar, Y. Pu, S. M. A. Augustino, X. Xue, F. Xing, G. Gebreselassie, J. Mwacharo, Y. Ma and Q. Zhao	10.3390/genes11050494
**2020**	Human Plasmodium vivax diversity, population structure and evolutionary origin	PLoS Negl Trop Dis	V. Rougeron, E. Elguero, C. Arnathau, B. Acuña Hidalgo, P. Durand, S. Houze, A. Berry, S. Zakeri, R. Haque, M. Shafiul Alam, F. Nosten, C. Severini, T. Gebru Woldearegai, B. Mordmüller, P. G. Kremsner, L. González-Cerón, G. Fontecha, D. Gamboa, L. Musset, E. Legrand, O. Noya, T. Pumpaibool, P. Harnyuttanakorn, K. M. Lekweiry, M. Mohamad Albsheer, M. Mahdi Abdel Hamid, A. Boukary, J. F. Trape, F. Renaud and F. Prugnolle	10.1371/journal.pntd.0008072
**2020**	In silico analysis of likely pathogenic variants in human GGCX gene	Informatics in Medicine Unlocked	M. O. Hassan, D. A. Gassim, A. M. Albakrye, H. A. Elnasri and M. A. M. Khaier	https://doi.org/10.1016/j.imu.2020.100337
**2020**	Increased Prevalence of TG and TPO Mutations in Sudanese Children With Congenital Hypothyroidism	J Clin Endocrinol Metab	R. J. Bruellman, Y. Watanabe, R. S. Ebrhim, M. K. Creech, M. A. Abdullah, A. M. Dumitrescu, S. Refetoff and R. E. Weiss	10.1210/clinem/dgz297
**2020**	Insertion of an Alu Element in Thyroglobulin Gene as a Novel Cause of Congenital Hypothyroidism	Thyroid	R. Bruellman, Y. Watanabe, R. Shareef, M. A. Abdullah, A. Dumitrescu, B. S. Strauss, S. Refetoff and R. E. Weiss	10.1089/thy.2019.0636
**2020**	Investigation of the EIL/EIN3 Transcription Factor Gene Family Members and Their Expression Levels in the Early Stage of Cotton Fiber Development	Plants (Basel)	H. Salih, S. He, H. Li, Z. Peng and X. Du	10.3390/plants9010128
**2020**	Isolation of endophytic fungi from South African plants, and screening for their antimicrobial and extracellular enzymatic activities and presence of type I polyketide synthases	South African Journal of Botany	M. A. Abdalla, A. O. Aro, D. Gado, A. K. Passari, V. K. Mishra, B. P. Singh and L. J. McGaw	https://doi.org/10.1016/j.sajb.2020.03.021
**2020**	Ixodid tick species and two tick-borne pathogens in three areas in the Sudan	Parasitol Res	Y. A. Shuaib, A. M. W. Elhag, Y. A. Brima, M. A. Abdalla, A. O. Bakiet, S. E. Mohmed-Noor, G. Lemhöfer, M. Bestehorn, S. Poppert, S. Schaper, G. Dobler, D. K. Bakkes and L. Chitimia-Dobler	10.1007/s00436-019-06458-9

**2020**	Madurella mycetomatis, the main causative agent of eumycetoma, is highly susceptible to olorofim	J Antimicrob Chemother	W. Lim, K. Eadie, M. Konings, B. Rijnders, A. H. Fahal, J. D. Oliver, M. Birch, A. Verbon and W. van de Sande	10.1093/jac/dkz529
**2020**	Madurella real-time PCR, a novel approach for eumycetoma diagnosis	PLoS Negl Trop Dis	A. Arastehfar, W. Lim, F. Daneshnia, W. W. J. van de Sande, A. H. Fahal, M. Desnos-Ollivier, G. S. de Hoog, T. Boekhout and S. A. Ahmed	10.1371/journal.pntd.0007845
**2020**	Molecular and Phenotypic Characterization of Nannizzia (Arthrodermataceae)	Mycopathologia	K. Dukik, G. S. de Hoog, J. B. Stielow, J. Freeke, B. G. van den Ende, V. A. Vicente, S. B. J. Menken and S. A. Ahmed	10.1007/s11046-019-00336-9
**2020**	Molecular characterization and diagnostic investigations of rabies encephalitis in camels (Camelus dromedaries) in Oman: a retrospective study	Trop Anim Health Prod	M. S. Ahmed, M. H. Body, M. S. El-Neweshy, A. L. AH, M. Al-Abdawani, H. A. Eltahir and A. L. MG	10.1007/s11250-020-02239-0
**2020**	Molecular characterization and susceptibility screening for methicillin-resistant Staphylococcus aureus reveals the dominant clones in a tertiary care hospital in Al Qassim, Saudi Arabia	Int J Health Sci (Qassim)	K. B. Said, A. N. Aljarbou, M. S. Alorainy, E. M. A. Saeed and K. M. Hassan	PMID: 31983916
**2020**	New codon 198 β-tubulin polymorphisms in highly benzimidazole resistant Haemonchus contortus from goats in three different states in Sudan	Parasit Vectors	K. M. Mohammedsalih, J. Krücken, A. Khalafalla, A. Bashar, F. R. Juma, A. Abakar, A. A. H. Abdalmalaik, G. Coles and G. von Samson-Himmelstjerna	10.1186/s13071-020-3978-6
**2020**	Optimization of pyrrolizine-based Schiff bases with 4-thiazolidinone motif: Design, synthesis and investigation of cytotoxicity and anti-inflammatory potency	European Journal of Medicinal Chemistry	A. M. Shawky, M. A. S. Abourehab, A. N. Abdalla and A. M. Gouda	https://doi.org/10.1016/j.ejmech.2019.111780
**2020**	Prevalence of 3.7 and 4.2 deletions in Sudanese patients with red cells hypochromia and microcytosis	BMC Res Notes	H. A. Osman, M. M. A. Hamid, R. B. Ahmad, M. Saleem and S. A. Abdallah	10.1186/s13104-020-4933-5
**2020**	Screening and mechanistic study of key sites of the hemagglutinin-neuraminidase protein related to the virulence of Newcastle disease virus	Poultry Science	C. Yan, H. Liu, Y. Jia, D. A. G. U. I. A. W. E. N. A. M. Prince-Theodore, M. Yang, F. E. Addoma Adam, J. Ren, X. Cao, X. Wang, S. Xiao, S. Zhang and Z. Yang	https://doi.org/10.1016/j.psj.2020.04.014
**2020**	Seroprevalence of measles, mumps, and rubella and genetic characterization of mumps virus in Khartoum, Sudan	Int J Infect Dis	O. Adam, A. Musa, A. Kamer, A. Sausy, E. Tisserand and J. M. Hübschen	10.1016/j.ijid.2019.11.019
**2020**	Subsistence strategy was the main factor driving population differentiation in the bidirectional corridor of the African Sahel	Am J Phys Anthropol	J. Nováčková, M. Čížková, M. G. Mokhtar, P. Duda, V. Stenzl, P. Tříska, Z. Hofmanová and V. Černý	10.1002/ajpa.24001
**2020**	Targeted deep amplicon sequencing of kelch 13 and cytochrome b in Plasmodium falciparum isolates from an endemic African country using the Malaria Resistance Surveillance (MaRS) protocol	Parasit Vectors	M. L'Episcopia, J. Kelley, D. Patel, S. Schmedes, S. Ravishankar, M. Menegon, E. Perrotti, A. M. Nurahmed, A. A. Talha, B. Y. Nour, N. Lucchi, C. Severini and E. Talundzic	10.1186/s13071-020-4005-7

**2020**	The Red Flour Beetle as Model for Comparative Neural Development: Genome Editing to Mark Neural Cells in Tribolium Brain Development	Methods Mol Biol	M. S. Farnworth, K. N. Eckermann, H. M. M. Ahmed, D. S. Mühlen, B. He and G. Bucher	10.1007/978-1-4939-9732-9_11
**2020**	Toxicity study and blood pressure–lowering efficacy of whey protein concentrate hydrolysate in rat models, plus peptide characterization	Journal of Dairy Science	F. A. Hussein, S. Y. Chay, S. B. M. Ghanisma, M. Zarei, S. M. Auwal, A. A. Hamid, W. Z. W. Ibadullah and N. Saari	https://doi.org/10.3168/jds.2019-17462
**2020**	Voriconazole resistance genes in Aspergillus flavus clinical isolates	Journal de Mycologie Médicale	F. Zaini, E. Lotfali, A. Fattahi, E. Siddig, S. Farahyar, E. Kouhsari and M. Saffari	https://doi.org/10.1016/j.mycmed.2020.100953
**2020**	Whey protein polymorphisms in Sudanese goat breeds	Trop Anim Health Prod	S. A. Rahmatalla, D. Arends, A. S. Ahmed, M. Reissmann and G. A. Brockmann	10.1007/s11250-019-02119-2

[a]The definition of “bioinformatics tools” in this study is identified by: Luscombe NM, Greenbaum D, Gerstein M: What is bioinformatics? A proposed definition and overview of the field. *Methods Inf Med* 2001, 40:346–358.

[b]Refer to the main text for inclusion and exclusion criteria.
